# Inhibition of PHD3 by salidroside promotes neovascularization through cell–cell communications mediated by muscle-secreted angiogenic factors

**DOI:** 10.1038/srep43935

**Published:** 2017-03-07

**Authors:** Jing Zhang, Vivi Kasim, Yu-Dan Xie, Can Huang, Julita Sisjayawan, Agnes Dwi Ariyanti, Xue-Song Yan, Xiao-Yan Wu, Cai-Ping Liu, Li Yang, Makoto Miyagishi, Shou-Rong Wu

**Affiliations:** 1 The Key Laboratory of Biorheological Science and Technology, Ministry of Education, College of Bioengineering, Chongqing University, Chongqing 400044, China; 2 The 111 Project Laboratory of Biomechanics and Tissue Repair, College of Bioengineering, Chongqing University, Chongqing 400044, China; 3 Molecular Composite Medicine Research Group, Biomedical Research Institute, National Institute of Advanced Industrial Science and Technology (AIST), Tsukuba 305-8566, Japan

## Abstract

Therapeutic angiogenesis has been considered as a potential strategy for treating peripheral artery diseases including hind-limb ischemia (HLI); however, no effective drug-based treatment is currently available. Here we showed that intramuscular administration of salidroside, an active compound of Chinese herb *Rhodiola*, could robustly enhance blood perfusion recovery by promoting neovascularization in HLI mice. We revealed that salidroside promoted skeletal muscle cell migration and paracrine function through inhibiting the transcriptional level of prolyl-hydroxylase domain 3 (PHD3) without affecting PHD1 and PHD2. Paracrine signals from salidroside-treated skeletal muscle cells enhanced endothelial and smooth muscle cells migration, while inhibition of FGF2/FGF2R and PDGF-BB/PDGFR-β pathways abolished this effect, as well as neovascularization in HLI mice. Furthermore, we elucidated that salidroside inhibition on PHD3 might occur through estrogen receptor alpha (ERα). Together, our findings highlights the potential application of salidroside as a novel pharmalogical inhibitor of ERα/PHD3 axis for therapeutic angiogenesis in HLI diseases.

Peripheral artery disease (PAD) is caused by obstructions in the arteries due to atherosclerosis, which limits blood supply to organs besides heart and brain^1^. PAD affects more than 5% of people aged 40 years or more, and its prevalence increases with age^2^,^3^. PAD most commonly caused gradual reduction of blood supply to the lower extrimity, resulting in hind-limb ischemia (HLI)^4^. Patients with critical limb ischemia (CLI), which is the most severe manifestation of PAD, typically have pain even at rest, ulcers, gangrene, poor prognosis, and a quality of life comparable to patients with advanced cancers^3^,^5^,^6^. Although the limb salvage rate by using catheter-based revascularization in correctly selected patients is more than 75% in one year, a significant number of patients are deemed poor or no-option candidates for revascularization, and unfortunately, this group has an extremely poor prognosis, with a 40% 1-year major amputation rate and a 20% 1-year mortality^2^,^3^,^7^,^8^,^9^.

In response to hypoxic stress, skeletal muscle cells have been shown to secrete paracrine and autocrine signals which could mediate cell–cell communication^2^,^10^. Cell–cell communication, either through direct cell–cell contact, transfer of secreted paracrine molecules, or exchange of extracellular vesicles, had been shown to play crucial roles in pathological responses including angiogenesis^11^. Therefore, skeletal muscle cells have currently become an attractive therapeutic target for PAD, and inducing therapeutic angiogenesis through activating skeletal muscle cells paracrine function is considered one of the most promising treatments for PAD^10^,^12^,^13^,^14^,^15^.

Regardless of the attempts made to develop strategies for therapeutic angiogenesis by using angiogenic factors in the form of protein, gene, or stem cell-based therapies, no effective drug-based treatment is currently available^14^,^16^,^17^,^18^,^19^,^20^,^21^,^22^. *Rhodiola*, a plant growing at high altitude and has been used since centuries in Chinese and Tibetan traditional medicine, has been known for its functions in enhancing adaptation to high-altitude and hypoxic condition, partly through the induction of EPO expression, as well as cell survival^23^,^24^. Recent studies have revealed that salidroside (2-[4-hydroxyphenyl]ethyl beta-d-glucopyranoside), which now could be artificially synthesized, is the main compound responsible for the therapeutic effect of *Rhodiola*^25^,^26^. However, whether or not salidroside exerts therapeutic angiogenesis effects in ischemic diseases has not been elucidated yet. Moreover, the knowledge on its pharmacological mechanisms is still very limited.

Here, we identified prolyl-hydroxylase domain 3 (PHD3), a member of the PHD family, as a novel target of salidroside. Salidroside specifically suppresses the expression level of PHD3 in skeletal muscle cells through estrogen receptor alpha (ERα). Specific inhibition of PHD3 by salidroside in skeletal muscle cells promoted their mobility, and concomitantly, their expression and secretion of angiogenic factors, which in turn enhanced cell–cell communication between skeletal muscle cells and endothelial and/or smooth muscle cells. Finally, we found that intramuscular salidroside administration specifically inhibits PHD3 expressions in skeletal muscle cells, and led to effective neovascularization and blood perfusion recovery in a mouse model of HLI, indicating that salidroside might be a potential drug candidate for therapeutic angiogenesis in HLI.

## Results

### Salidroside improves blood perfusion recovery in the ischemic hind limb of a HLI mouse model

**Figure 1:**
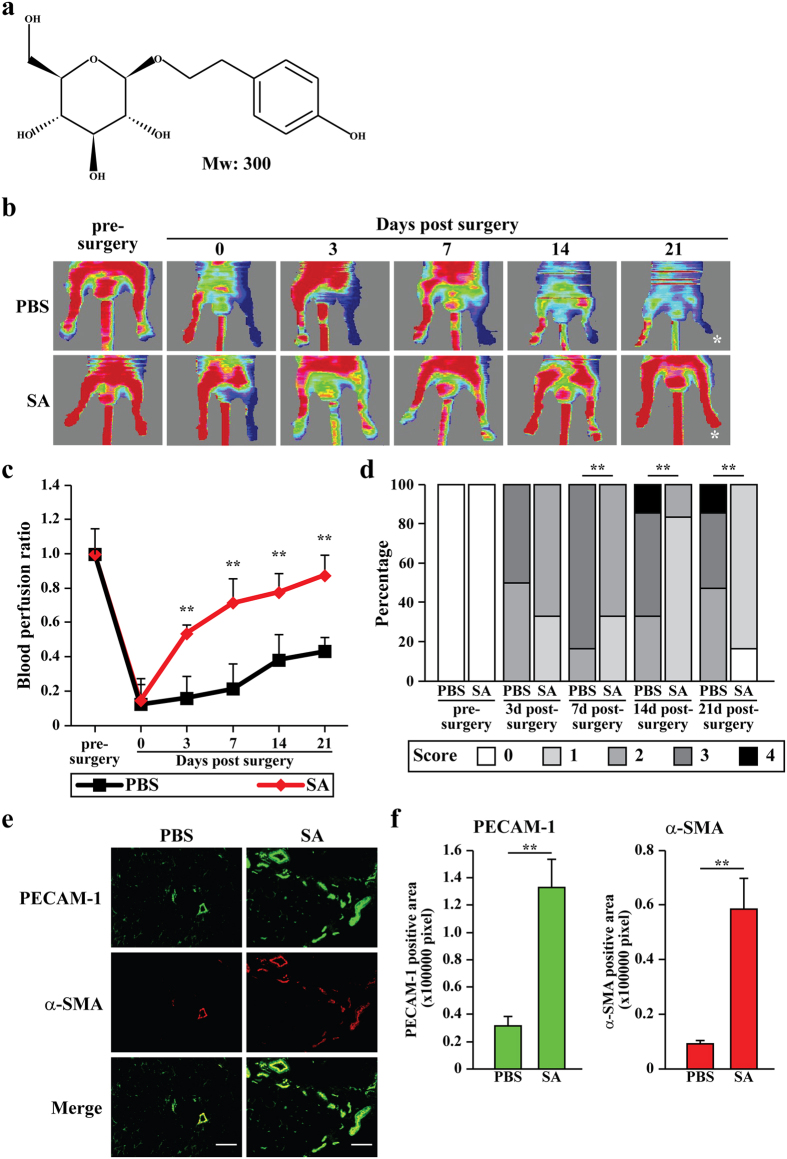
Salidroside promotes the blood perfusion recovery and formation of mature blood vessels in the ischemic hind limb of HLI mice. (**a**) Chemical structures of salidroside. (**b**,**c**) The blood perfusion in the ischemic hind limbs of HLI mice treated with salidroside or PBS at the indicated times: (**b**) representative of Laser Doppler Perfusion Images; (**c**) the blood perfusion ratio of ischemic hind limb to non-ischemic hind limb (n = 6–7 per group, **p < 0.01). (**d**) Assessment test of the ischemic hind limb morphologies of HLI mice treated by salidroside or PBS at indicated times (n = 6–7 per group, **p < 0.01 versus salidroside-treated group. 0 = no difference from the non-ischemic hind limb, 1 = mild discoloration, 2 = moderate discoloration, 3 = severe discoloration, subcutaneous tissue loss, or necrosis, and 4 = amputation). (**e**,**f**) Immunohistochemistry against PECAM-1 (green) and α-SMA (red) in the gastrocnemius muscle of the ischemic hind limbs of HLI mice treated by either salidroside or PBS at 21 days post-surgery: (**e**) representative images (scale bars: 100 μm); (**f**) quantification of PECAM-1 and α-SMA positive areas, Quantitative data were shown as means ± s.e.m of three independent experiments (**p < 0.01). SA: salidroside.

Neovascularization is one of the major pathological responses when cells are exposed to ischemic stress in order to guarantee sufficient oxygen supply. *Rhodiola* is known for its function in improving cellular adaptation to hypoxic conditions. Thus, we questioned whether salidroside (Fig. 1a), the main active compound of *Rhodiola*, could promote neovascularization in ischemic tissue. We first examined the effect of salidroside on blood perfusion recovery in the ischemic hind limbs of HLI-model mice. Intramuscular injection of salidroside (Supplementary Fig. 1a) resulted in blood perfusion recovery starting from day 3 post-surgery (Fig. 1b). As shown in Fig. 1c, when compared to the control group administered with phosphate-buffered saline (PBS), salidroside-treated HLI mice displayed a significantly higher blood perfusion recovery at 21 days post-surgery. Consistent with these results, ischemic damage assessment showed that at 21 days post-surgery, HLI mice treated with salidroside scored 0–1, while those treated with PBS mice scored 2–4 (Fig. 1d). Immunohistochemistry results showed that when compared to PBS-treated mice, salidroside-treated HLI mice displayed 4- and 6-fold increases in endothelial (platelet endothelial cell adhesion molecule 1 (PECAM-1)-positive) and smooth muscle (alpla-smooth muscle actin (α-SMA)-positive) cells, respectively, in the ischemic hind limb (Fig. 1e,f). The number of vessel structures doubly positive for PECAM-1 and α-SMA, which represent mature blood vessels, also showed a robust increase in the ischemic hind limb of salidroside-treated HLI mice (Fig. 1e, merge). Furthermore, we did not observe any obvious morphological changes in the liver, kidneys, spleen, and heart after a 2-month administration of salidroside (Supplementary Fig. 2). Together, these results showed that intramuscular salidroside treatment could effectively increase the blood perfusion recovery in the ischemic hind limbs of HLI mice, most plausibly owing to the increase of the number of mature blood vessels in the ischemic hind limb.

### Salidroside promotes mobility and paracrine function of skeletal muscle cells

**Figure 2:**
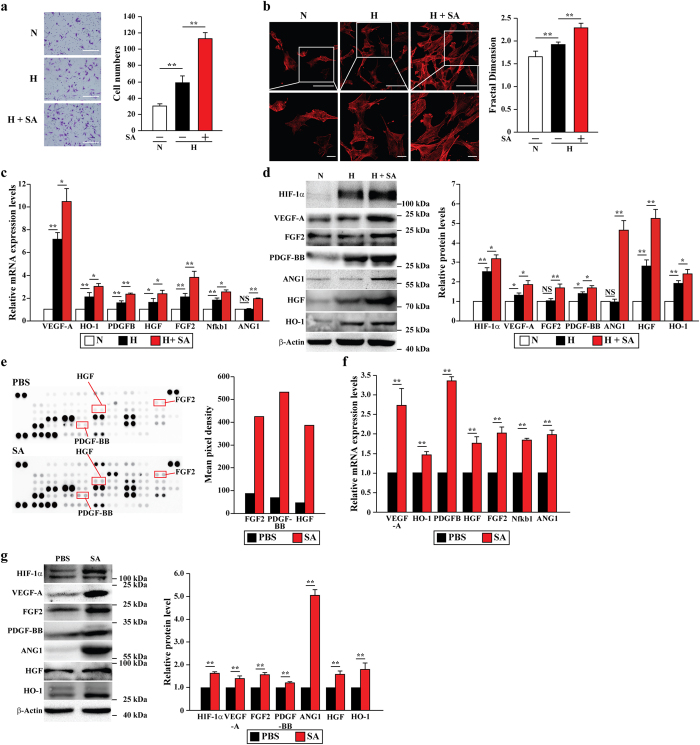
Salidroside enhances the mobility and paracrine function of skeletal muscle cells. (**a**) Transwell chamber assay showing the mobility of salidroside-treated C2C12 cells: representative images (left) (scale bars: 100 μm) and quantification of migrated cells (right) (**p < 0.01). (**b**) Morphological changes of F-actin in salidroside-treated C2C12 cells were examined by using phalloidin staining: representative images (left) and fractal dimension analysis (right) (**p < 0.01). Scale bars: 100 μm (upper panels) or 25 μm (lower panels). (**c**,**d**) The mRNA (**c**) and protein (**d**) expression levels of angiogenic factors in C2C12 cells treated with salidroside were analyzed by using quantitative RT-PCR (*p < 0.05, **p < 0.01, data were shown as relative value to the expression level in normoxia) and western blotting: representative images (left) and quantitative analysis (right) (NS: not significant, *p < 0.05, **p < 0.01). (**e**) Representative images (left) and quantification (right) of angiogenic factors protein array from the medium of C2C12 cells treated with PBS or salidroside. The protein array was repeated twice. (**f**,**g**) The mRNA (**f**) and protein (**g**) expression levels of angiogenic factors in the gastrocnemius muscle of the ischemic hind limbs of HLI mice treated with salidroside were analyzed by using quantitative RT-PCR (**p < 0.01, data were shown as relative value to the expression level in the ischemic hind limbs of HLI mice treated with PBS) and western blotting: representative images (left) and quantitative analysis (right) (**p < 0.01). Quantitative data (**a**–**d**,**f**,**g**) were shown as means ± s.e.m of three independent experiments. N: normoxia, H: hypoxia, SA: salidroside.

Skeletal muscle cells play critical roles in angiogenesis as they express and secrete angiogenic factors^10^. To investigate the effect of salidroside on skeletal muscle cells, we first analyzed the effect of salidroside on murine myoblast cell line C2C12 proliferation. Ki67 staining results revealed that salidroside promoted C2C12 cells proliferation (Supplementary Fig. 3a). Although salidroside slightly enhanced the resistance of C2C12 cells to apoptosis, the number of apoptotic cells under hypoxic condition was not significant (Supplementary Fig. 3b). On the other hand, as evident from the transwell chamber assay (Fig. 2a) and scratch assay (Supplementary Fig. 3c,d) results, the mobility of the C2C12 cells under hypoxia was conspicuously enhanced by salidroside treatment. Consistent with this, phalloidin staining results showed a significant increase in pseudopodia and polymerization of G-actin to F-actin in the salidroside-treated C2C12 cells (Fig. 2b).

Next, we further investigated whether or not salidroside affects the paracrine function of skeletal muscle cells cultured under hypoxia. As shown in Fig. 2c, the mRNA expression levels of angiogenic factors such as vascular endothelial growth factor A (VEGF-A), heme oxygenase 1 (HO-1), platelet-derived growth factor B (PDGFB), hepatocyte growth factor (HGF), fibroblast growth factor 2 (FGF2), nuclear factor kappa b1 (Nfkb1), and angiopoietin 1 (ANG1) were upregulated when C2C12 cells were treated with salidroside. The protein expression levels of these factors, as well as HIF-1α, also showed same tendency (Fig. 2d). Furthermore, protein array results showed a robust increase in the amounts of various angiogenic factors, including PDGF-BB, FGF2 and HGF, in the culture medium of salidroside-treated C2C12 cells (Fig. 2e) compared to that of the PBS-treated cells. These findings were corroborated by the increased mRNA and protein levels of angiogenic factors in the gastrocnemius muscle of the ischemic hind limbs of the HLI mice (Fig. 2f,g, respectively). Together, these results indicated that salidroside greatly enhances the mobility of skeletal muscle cells, and their expression and secretion of angiogenic factors.

### Salidroside enhances the mobility of endothelial and smooth muscle cells via skeletal muscle cell paracrine signaling

**Figure 3:**
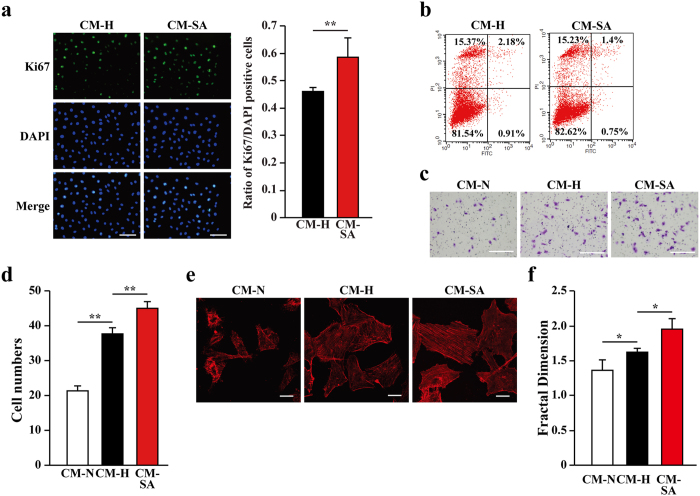
Salidroside-induced skeletal muscle cells secretome enhances the proliferation and mobility of endothelial cells. (**a**) The proliferation of HUVECs cultured with CM-SA was analyzed using Ki67 staining. The nuclei were stained with DAPI. (Left) representative images (Scale bars: 100 μm); (right) ratio of Ki67 positive cells to DAPI positive cells (**p < 0.01). (**b**) The percentage of apoptotic cells in HUVECs cultured with CM-SA was analyzed using Annexin V-FITC/PI staining and FACS analysis. (**c**,**d**) The mobility of HUVECs cultured with CM-SA was examined by using transwell chamber assay: (**c**) representative images (scale bars: 100 μm) and (**d**) quantification of migrated cells (**p < 0.01). (**e**,**f**) Morphological changes of F-actin were examined by using phalloidin staining: (**e**) representative images (scale bars: 25 μm); (**f**) quantification analysis of fractal dimension (*p < 0.05). CM-SA: conditioned medium from salidroside-treated C2C12 cells cultured under hypoxia; CM-N and CM-H: conditioned medium from PBS-treated C2C12 cells cultured under normoxia or hypoxia, respectively. All experiments were done in hypoxic condition. Quantitative data (**a**,**d**,**f**) were shown as means ± s.e.m of three independent experiments.

The fact that salidroside enhanced the secretion of angiogenic factors from skeletal muscle cells suggested that salidroside might affect endothelial and smooth muscle cells through skeletal muscle cell-secreted angiogenic factors. To examine this possibility, we prepared conditioned media containing the secretome of C2C12 cells treated with PBS and cultured under normoxia (“CM-N”); and treated with PBS or salidroside and cultured under hypoxia (“CM-H” and “CM-SA”, respectively) (Supplementary Fig. 4a). It is noteworthy that, as shown by the results of mass spectrometry in Supplementary Fig. 4b–f, as the cells were washed with PBS and the medium was changed to a fresh one after being treated with salidroside, the CM-SA did not contain salidroside anymore. Indeed, CM-SA could induce the growth of HUVECs (Fig. 3a) and MOVAS cells (Supplementary Fig. 5a), while its effect on the apoptosis resistance of these cells was more subtle: as compared to CM-H, CM-SA only slightly decreased the number of apoptotic cells (Fig. 3b and Supplementary Fig. 5b for HUVECs and MOVAS cells, respectively).

We next examined the effect of CM-SA on the migration potential of HUVECs and MOVAS cells. As indicated by the transwell chamber assay and scratch assay results, CM-SA robustly increased the mobility of HUVECs (Fig. 3c,d and Supplementary Fig. 5c,d, respectively), most likely by inducing F-actin polymerization as indicated by phalloidin staining (Fig. 3e,f). The mobility-enhancing effect of CM-SA on MOVAS cells was even much more significant, as they showed more than 2-fold higher migration in CM-SA than in CM-H (Supplementary Fig. 5e–h). Moreover, phalloidin staining demonstrated increased pseudopodia and actin filaments in MOVAS cells cultured in CM-SA as compared to CM-N and CM-H (Supplementary Fig. 5i,j). Thus, these results showed that salidroside exerts its effect on the mobility and proliferation potential of endothelial and smooth muscle cells through skeletal muscle cell-secreted angiogenic factors.

### Salidroside affects the mobility of endothelial and smooth muscle cells through FGF2/FGF2R and PDGF-BB/PDGFR-β axis

**Figure 4:**
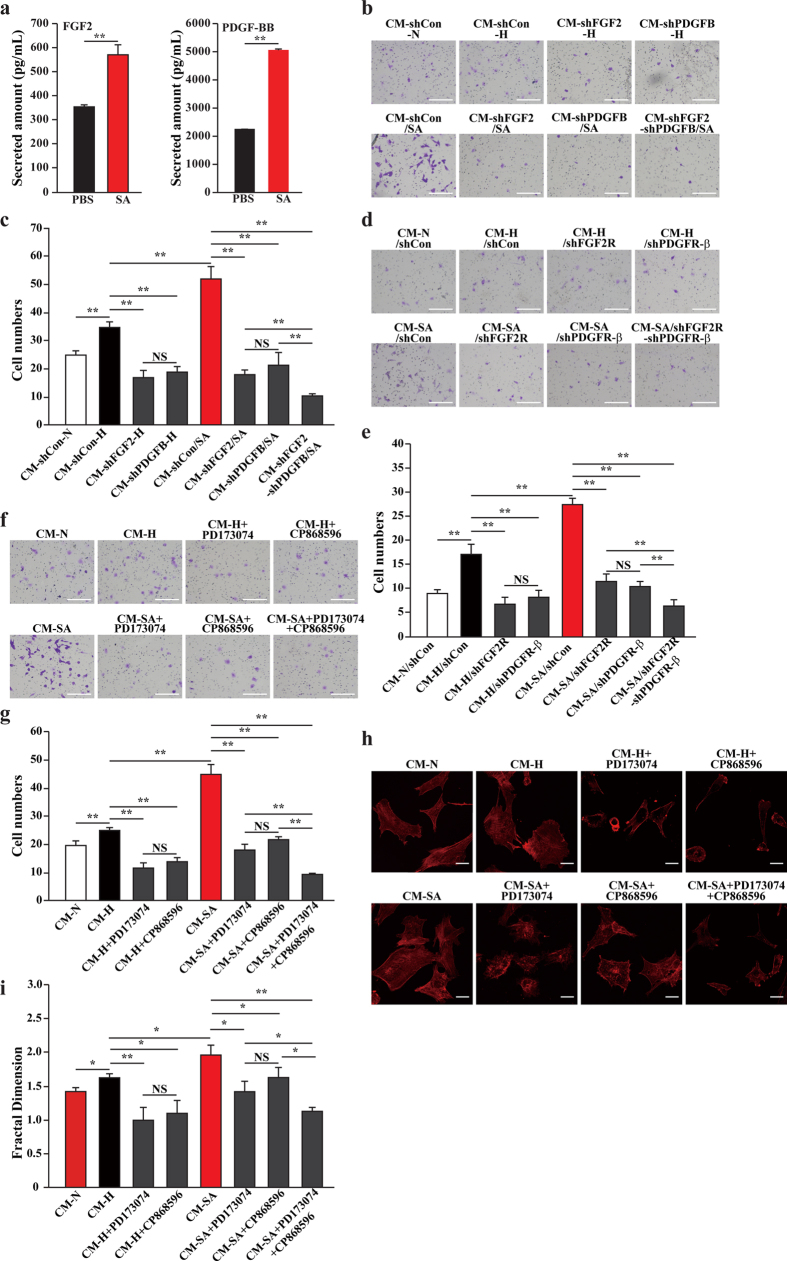
Skeletal muscle cells-secreted FGF2 and PDGF-BB mediate salidroside-enhanced skeletal muscle–endothelial cells communications. (**a**) The amounts of secreted FGF2 (left) and PDGF-BB (right) in the medium of C2C12 cells treated with salidroside or PBS were analyzed using ELISA (**p < 0.01). (**b**,**c**) The mobility of HUVECs cultured with conditioned media collected from FGF2- or PDGFB-silenced C2C12 cells and treated with salidroside: (**b**) representative images and (**c**) quantification of migrated cells (NS: not significant, **p < 0.01). (**d**,**e**) The mobility of FGF2R- or PDGFR-β-silenced HUVECs cells cultured with CM-SA were analyzed by using transwell chamber assay: (**d**) representative images and (**e**) quantification of migrated cells (NS: not significant, **p < 0.01). (**f**,**g**) The mobility of HUVECs cultured with CM-SA and PD173074, CP868596 or both of them were analyzed by using transwell chamber assay: (**f**) representative images and (**g**) quantification of migrated cells (NS: not significant, **p < 0.01). (**h**–**i**) Morphological changes of F-actin were examined by using phalloidin staining: (**h**) representative images; (**i**) quantitation analysis of fractal dimension (NS: not significant, *p < 0.05, **p < 0.01). SA: salidroside; CM-N and CM-H: conditioned medium from PBS-treated C2C12 cells cultured under normoxia or hypoxia, respectively; CM-SA: conditioned medium from salidroside-treated C2C12 cells cultured under hypoxia; CM-shCon-N and CM-shCon-H: conditioned medium from shCon-transfected, PBS-treated C2C12 cells cultured under normoxia or hypoxia, respectively; CM-shFGF2-H and CM-shPDGFB-H: conditioned medium from FGF2- or PDGFB-silenced C2C12 cells cultured under hypoxia, respectively; CM-shCon/SA, CM-shFGF2/SA, CM-shPDGFB/SA and CM-shFGF2-shPDGFB/SA conditioned medium from shCon-transfected, FGF2-silenced, PDGFB-silenced or FGF2 and PDGFB-silenced, salidroside-treated C2C12 cells cultured under hypoxia, respectively; CM-N/shCon, CM-H/shCon, CM-H/shFGF2R, CM-H/shPDGFR-β, CM-SA/shCon, CM-SA/shFGF2R, CM-SA/shPDGFR-β and CM-SA/shPDGFR-β-shFGF2R: HUVECs transfected with shCon, shFGF2R or shPDGFR-β or both of them and cultured with CM-N, CM-H or CM-SA. Scale bars: 100 μm (**b**,**d**,**f**) or 25 μm (**h**). All experiments were done in hypoxic condition. Quantitative data (**a**,**c**,**e**,**g**,**i**) were shown as means ± s.e.m of three independent experiments.

Previous studies have reported that FGF2 and PDGF-BB play critical roles in promoting the proliferation and mobility of both endothelial and smooth muscle cells^27^,^28^,^29^,^30^,^31^,^32^,^33^,^34^. The results of protein array also showed that the amounts of FGF2 and PDGF-BB, especially, FGF2—which was almost undetectable in the medium of PBS-treated C2C12 cells—were significantly higher in the medium of salidroside-treated C2C12 cells (Fig. 2e), and enzyme-linked immunosorbent assay (ELISA) results were consistent with these findings (Fig. 4a). Thus, although the effect of salidroside on neovascularization is most plausibly a synergistic effect of multiple angiogenic factors, here we focus on the role of PDGF-BB and FGF2.

To determine the roles of FGF2 and PDGF-BB secreted from salidroside-treated skeletal muscle cells on the migration of HUVECs and MOVAS cells, we first silenced the expressions of FGF2 and PDGFB in C2C12 cells by using shRNA expression vectors targeting them prior to treatment by using salidroside. Two shRNA vectors targeting different target sites were used for each gene to eliminate the nonspecific effect (Suppplementary Fig. 6a). Transwell chamber assay results showed that migration of HUVECs (Fig. 4b upper panels and Fig. 4c) and MOVAS cells (Supplementary Fig. 6b,c) induced by conditioned medium collected from FGF2- and PDGFB-silenced C2C12 cells were significantly lower than control (*i.e.*, conditioned medium collected from C2C12 cells transfected with shCon and cultured in hypoxia, CM-shCon-H). Furthermore, the effect of salidroside-induced C2C12 cells’ secretome on HUVECs (Fig. 4b lower panels and Fig. 4c) and MOVAS cells (Supplementary Fig. 6b,c) migration was also cancelled when the expression of FGF2 and PDGFB in the C2C12 cells were suppressed, indicating that salidroside induces HUVECs and MOVAS cells migration through, at least partly, FGF2 and PDGF-BB secreted by salidroside-treated skeletal muscle cells.

Next we examined the roles of FGF2 receptor (FGF2R) and PDGF receptor beta (PDGFR-β) on the migration of HUVECs and MOVAS cells induced by CM-SA, we firstly silenced the expressions of FGF2R and PDGFR-β in HUVECs and MOVAS by using two shRNA expression vectors targeting different target sites for each gene to eliminate the nonspecific effect (Supplementary Fig. 6d,e). Transwell chamber assay results showed that migration of HUVECs (Fig. 4d,e) and MOVAS cells (Supplementary Fig. 6f,g) induced by salidroside-treated skeletal muscle cells’ secretomes were significantly inhibited when FGF2R and PDGFR-β were silenced. To further confirm the roles of FGF2/FGF2R and PDGF-BB/PDGFR-β pathway on the migration of HUVECs and MOVAS cells, we used FGF2R inhibitor PD173074, PDGFR inhibitor CP868596 or a combination of both of them. To validate the effective inhibition of FGF2R and PDGFR, we examined the expression levels of PI3K and EPO, downstream targets of FGF2/FGF2R and PDGF-BB/PDGFR-β pathway, respectively (Supplementary Fig. 7a,b). The expressions of PI3K and EPO were significantly downregulated when PD173074 and CP868596 were added into the culture medium, respectively, indicating that the FGF2/FGF2R and PDGF-BB/PDGFR-β signaling pathways were successfully blocked.

Addition of PD173074 and CP868596 significantly inhibited the mobility of HUVECs (Fig. 4f upper panels and Fig. 4g) and MOVAS cells (Supplementary Fig. 7c upper panels) induced by salidroside-treated skeletal muscle cells’ secretome as demonstrated by the transwell chamber assay results. Furthermore, blocking either the FGF2R or PDGFR-β reduced the HUVECs migration induced by CM-SA by approximately 3-fold, while blocking of both pathways resulted in a ~7-fold reduction (Fig. 4f lower panels and Fig. 4g). The effects of these inhibitors on CM-SA-induced migration in MOVAS cells were even more pronounced; inhibition of either FGF2R or PDGFR-β resulted in a ~13-fold reduction in migrated cells, and simultaneous inhibition of both pathways almost totally abolished migration (Supplementary Fig. 7c lower panels). These effects were further confirmed by using scratch assay (Supplementary Fig. 7d–g). Furthermore, consistent with these, phalloidin staining revealed that polymerization of F-actin induced by CM-SA was conspicuously inhibited when the FGF2R or PDGFR-β were blocked (Fig. 4h,i for HUVECs and Supplementary Fig. 7h,i for MOVAS cells). Together, these results revealed the pivotal roles of the FGF2/FGF2R and PDGF-BB/PDGFR-β pathways in mediating the communication between skeletal muscle cells and endothelial and/or smooth muscle cells and in inducing endothelial and/or smooth muscle cells migration; and that salidroside could induce the migration of endothelial and/or smooth muscle cells through skeletal muscle cells by, at least, activating these pathways.

### The FGF2/FGF2R and PDGF-BB/PDGFR-β pathways mediate the therapeutic angiogenesis effect of salidroside in the HLI mouse model

**Figure 5:**
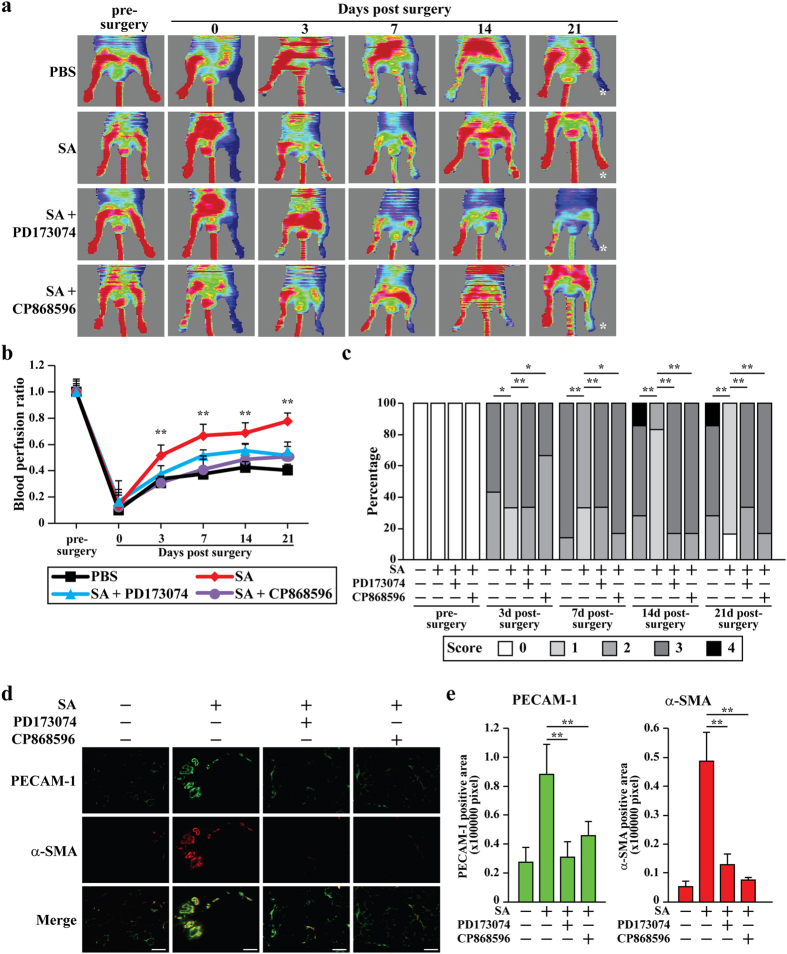
Salidroside promotes neovascularization in the ischemic hind limb of HLI mice through FGF2/FGF2R and PDGF-BB/PDGFR-β pathways. (**a**,**b**) The blood perfusion in the ischemic hind limbs of HLI mice administered with salidroside and PD173074 or CP868596: (**a**) representative of Laser Doppler Perfusion Images; (**b**) the blood perfusion ratio of ischemic hind limb to non-ischemic hind limb (n = 6–7 per group, **p < 0.01 versus salidroside-treated group). (**c**) Assessment test of the ischemic hind limb morphologies of HLI mice treated with salidroside and PD173074 or CP868596 at indicated times (n = 6–7 per group, *p < 0.05, **p < 0.01 versus salidroside-treated group. 0 = no difference from the non-ischemic hind limb, 1 = mild discoloration, 2 = moderate discoloration, 3 = severe discoloration, subcutaneous tissue loss, or necrosis, and 4 = amputation). (**d**,**e**) Immunohistochemistry against PECAM-1 (green) and α-SMA (red) in the gastrocnemius muscle of the ischemic hind limbs of HLI mice treated with salidroside and PD173074 or CP868596 at 21 days post-surgery: (**d**) representative images (scale bars: 100 μm); (**e**) quantification of PECAM-1 and α-SMA positive areas. Quantitative data were shown as means ± s.e.m of three independent experiments (**p < 0.01). SA: salidroside.

The roles of the FGF2/FGF2R and PDGF-BB/PDGFR-β pathways in the therapeutic angiogenesis effect of salidroside were further examined in the HLI mouse model by administering both salidroside and FGF2R or PDGFR-β inhibitors to the HLI mice (Supplementary Fig. 1b). Administration of salidroside gradually recovered the blood perfusion from 3 days post-surgery; however, administration of PD173074 or CP868596 clearly blocked this effect (Fig. 5a). Consistently, quantification results also revealed that at the end of the experiment, the blood perfusion ratio of ischemic hind limb to non-ischemic hind limbs of HLI mice treated with salidroside/PD173074 or salidroside/CP868596 only reached approximately 40%, which were significantly lower than that of salidroside-treated group (around 80%) (Fig. 5b). Accordingly, the therapeutic effect of salidroside on the ischemic damage was diminished by the administration of PD173074 or CP868596 (Fig. 5c). Furthermore, both agents robustly inhibited the salidroside-induced increase of PECAM-1- and α-SMA-positive cells in the ischemic hind limb to approximately the control level (Fig. 5d,e). Consistent with these findings, vasculature structures doubly positive for PECAM-1 and α-SMA were hardly observed when PD173074 and CP868596 were administered (Fig. 5d, merge). These facts further demonstrated that FGF2/FGF2R and PDGF-BB/PDGFR-β pathways play pivotal roles in salidroside-induced therapeutic angiogenesis in HLI mice.

### Specific inhibition of PHD3 mediates the effects of salidroside on skeletal muscle cells

**Figure 6:**
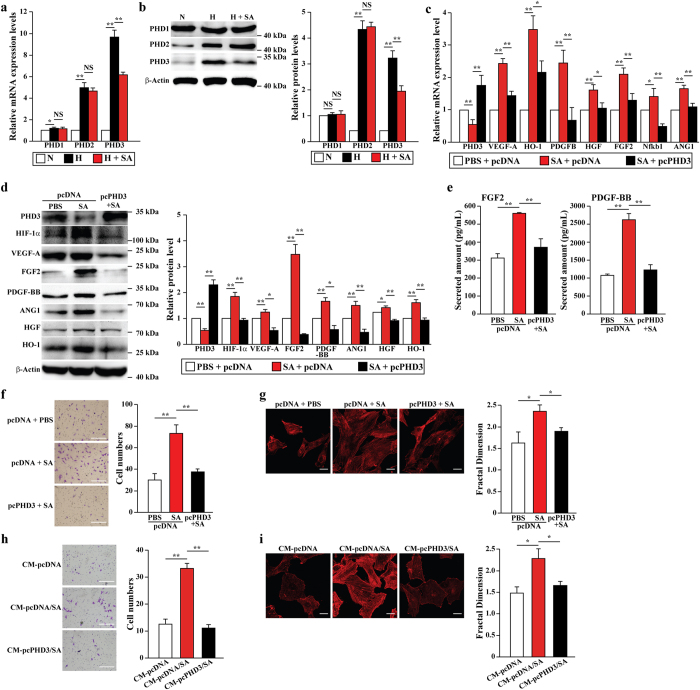
Salidroside promotes the mobility and paracrine function of skeletal muscle cells through specific inhibition of PHD3. (**a**,**b**) The mRNA (**a**) and protein (**b**) expression levels of PHDs family in C2C12 cells treated with salidroside were analyzed using quantitative RT-PCR (NS: not significant, *p < 0.05, **p < 0.01) and western blotting: representative images (left) and quantification (right) (NS: not significant, **p < 0.01). (**c**,**d**) The mRNA (**c**) and protein (**d**) expression levels of angiogenic factors in PHD3-overexpressing C2C12 cells treated with salidroside were analyzed using quantitative RT-PCR (*p < 0.05, **p < 0.01) and western blotting: representative images (left) and quantitative analysis (right) (*p < 0.05, **p < 0.01). (**e**) The amount of FGF2 (left) and PDGF-BB (right) in the medium collected from PHD3-overexpressing C2C12 cells treated with salidroside were analyzed by using ELISA (**p < 0.01). (**f**) The mobility of PHD3-overexpressing C2C12 cells treated with salidroside were analyzed by using transwell chamber assay: representative images (left) and quantification of migrated cells (right) (**p < 0.01). (**g**) Morphological changes of F-actin were examined by using phalloidin staining: representative images (left); quantification analysis of fractal dimension (right) (*p < 0.05). (**h**) The mobility of HUVECs cultured with CM-pcPHD3/SA were analyzed by using transwell chamber assay: representative images (left) and quantification of migrated cells (right) (**p < 0.01). (**i**) Morphological changes of F-actin were examined by using phalloidin staining: representative images (left); quantification analysis of fractal dimension (right) (*p < 0.05). Scale bars: 100 μm (**f**) or 25 μm (**g**,**i**). N: normoxia; H: hypoxia; SA: salidroside; CM-pcDNA, CM-pcDNA/SA and CM-pcPHD3/SA: conditioned medium from pcDNA3.1 (+)-transfected or pcPHD3-transfected C2C12 cells treated with PBS or salidroside. Quantitative data (**a**–**i**) were shown as means ± s.e.m of three independent experiments.

Previous studies had shown that PHD family members play critical roles in angiogenesis as they regulate the expression of a number of angiogenic factors^35^,^36^,^37^. Thus, we next examined the effect of salidroside on the PHD family. To our surprise, salidroside specifically downregulated PHD3 mRNA (Fig. 6a). Accordingly, the protein level of PHD3, but not PHD1 and PHD2, was suppressed by salidroside (Fig. 6b). Furthermore, the inhibitory effect of salidroside on PHD3 was dose-dependent (Supplementary Fig. 8a). These results demonstrated that PHD3 might be a specific target of salidroside.

To confirm the role of PHD3 in skeletal muscle cells, we first examined the effect of PHD3 silencing on the expression of angiogenic factors. The silencing effect of the vectors shPHD3–1 and shPHD3–2 was specific, as they did not affect the expression levels of PHD1 and PHD2 (Supplementary Fig. 8b,c) while they effectively suppressed PHD3 expression with more than 80% (Supplementary Fig. 8d,e). Consistent with the results of salidroside treatment, PHD3 silencing resulted in the robust upregulation of angiogenic factors expressions, and the accumulation of HIF-1α protein (Supplementary Fig. 8d,e). Furthermore, PHD3 silencing significantly induced the mobility of C2C12 cells, as shown by scratch and transwell assays, most likely by enhancing F-actin polymerization (Supplementary Fig. 8f–h). In contrast, PHD3 overexpression suppressed the mRNA and protein expression levels of the angiogenic factors (Supplementary Fig. 9a–c). In addition, PHD3 overexpression supressed the migration of C2C12 cells, as shown by transwell assays, most likely by enhancing F-actin polymerization (Supplementary Fig. 9d,e). Thus, these results demonstrated that the pivotal role of PHD3 in the expression of angiogenic factors and skeletal muscle cells migration.

To examine whether the effects of salidroside on the expression and secretion of angiogenic factors, especially FGF2 and PDGF-BB, are PHD3-dependent, we overexpressed PHD3 in the C2C12 cells and treated them with salidroside. As shown in Fig. 6c,d, PHD3 overexpression diminished the effect of salidroside on the mRNA and protein expression of angiogenic factors. Furthermore, the secretion of FGF2 and PDGF-BB, which was upregulated by salidroside, was suppressed to nearly the control levels when PHD3 was overexpressed (Fig. 6e). In consistence herewith, PHD3 overexpression attenuated the effect of salidroside on C2C12 cells mobility—F-actin polymerization was suppressed and migration was reduced by nearly 50% (Fig. 6f,g and Supplementary Fig. 10a,b).

Given that PHD3 overexpression inhibited the salidroside-induced secretion of FGF2 and PDGF-BB (Fig. 6e), we predicted that it would also negatively regulate the effect of salidroside on the mobility of endothelial and smooth muscle cells through FGF2/FGF2R and PDGF-BB/PDGFR-β pathways. As expected, when compared to cells cultured with conditioned medium obtained from C2C12 cells transfected with control plasmid and treated with salidroside (CM-pcDNA/SA), the mobility of HUVECs cultured with conditioned medium obtained from PHD3-overexpressing C2C12 treated with salidroside (CM-pcPHD3/SA), was largely suppressed (Fig. 6h and Supplementary Fig. 10c,d). Consistently, F-actin polymerization was also hampered (Fig. 6i). Similarly, the mobility and F-Actin polymerization level of MOVAS cells were significantly reduced when cultured with CM-pcPHD3/SA (Supplementary Fig. 10e–j). Taken together, these results demonstrated that salidroside affects the paracrine signaling such as FGF2 and PDGF-BB as well as the mobility of skeletal muscle cells through inhibiting PHD3, and subsequently, affects endothelial and smooth muscle cells.

### Salidroside exerts therapeutic angiogenesis effect through inhibition of PHD3

**Figure 7:**
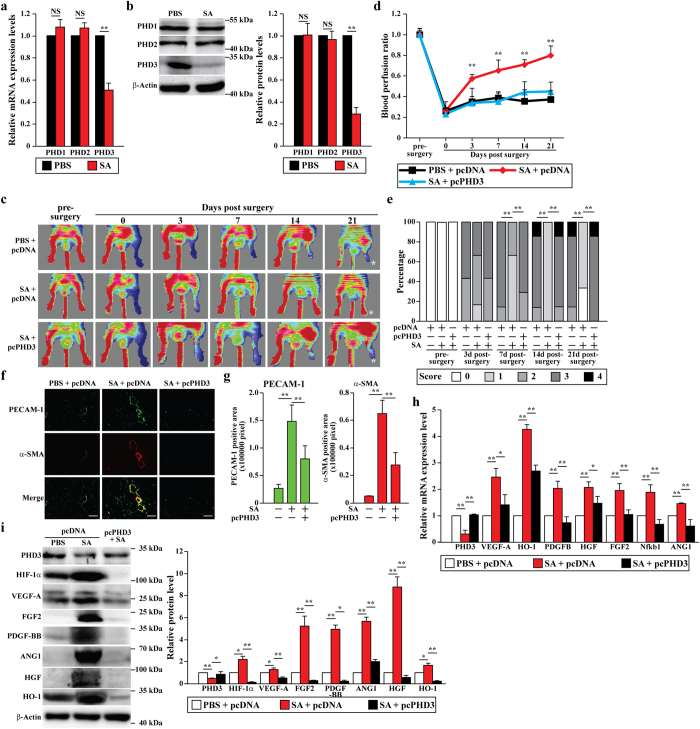
Salidroside specific inhibitory effect on PHD3 promotes neovascularization in the HLI mice. (**a**,**b**) The mRNA (**a**) and protein (**b**) expression levels of PHDs family in the gastrocnemius muscle of the ischemic hind limbs of HLI mice treated with salidroside were analyzed by using quantitative RT-PCR (NS: not significant, **p < 0.01) and western blotting: representative images (left) and quantitative analysis (right) (NS: not significant, **p < 0.01). (**c**,**d**) The blood perfusion in the ischemic hind limbs of HLI mice administered with salidroside and pcPHD3 or pcDNA3.1(+) plasmid: (**c**): representative of Laser Doppler Perfusion Images; (**d**): the blood perfusion ratio of ischemic hind limb to non-ischemic hind limb (n = 6–7 per group, **p < 0.01). (**e**) Assessment test of the ischemic hind limb morphologies of HLI mice treated by salidroside and pcPHD3 or pcDNA3.1(+) plasmid at indicated times (n = 6–7 per group, **p < 0.01 versus salidroside-treated group. 0 = no difference from the non-ischemic hind limb, 1 = mild discoloration, 2 = moderate discoloration, 3 = severe discoloration, subcutaneous tissue loss, or necrosis, and 4 = amputation). (**f**,**g**) Immunohistochemistry against PECAM-1 (green) and α-SMA (red) in the gastrocnemius muscle of the ischemic hind limbs of HLI mice administered with salidroside and pcPHD3 or pcDNA3.1(+) plasmid at 21 days post-surgery: (**f**) representative images; (**g**) quantification of PECAM-1 and α-SMA positive areas (**p < 0.01). (**h**,**i**) The mRNA (**h**) and protein (**i**) expression levels of PHD3 and angiogenic factors in the gastrocnemius muscle of the ischemic hind limbs of HLI mice administered with salidroside and pcPHD3 or pcDNA3.1(+) were analyzed by using quantitative RT-PCR (*p < 0.05, **p < 0.01) and western blotting: representative images (left) and quantitative analysis (right) (*p < 0.05, **p < 0.01), respectively. pcDNA: pcDNA3.1(+); SA: salidroside. Scale bars: 100 μm. Quantitative data (**a**,**b**,**g**,**h**,**i**) were shown as means ± s.e.m of three independent experiments.

Next, we examined PHD3 expression in salidroside-treated HLI mice. Consistent with the *in vitro* results, inhibition of PHD3, but not PHD1 and PHD2, was observed in the ischemic muscle tissue obtained from salidroside-treated HLI mice (Fig. 7a,b).

The above results prompted us to test whether salidroside-induced angiogenesis and the subsequent blood perfusion recovery in the ischemic hind limb was mediated by PHD3. We performed intramuscular injection of salidroside and pcPHD3 vector into the ischemic hind limb of HLI mice (the administration schedule was shown Supplementary Fig. 1c), and found that PHD3 overexpression significantly decreased salidroside-induced blood perfusion recovery (Fig. 7c). Furthermore, as shown in Fig. 7d, while salidroside administration induced blood perfusion recovery starting from day 3 post-surgery, the blood perfusion ratio in the ischemic hind limbs of HLI mice administered with salidroside and pcPHD3 did not show a significant difference with that of the control group throughout the experiment. The result of ischemic damage assessment was also consistent with blood perfusion recovery (Fig. 7e). Similarly, PHD3 overexpression also abolished the increases in PECAM-1-positive, α-SMA-positive, and doubly positive vessel structures induced by salidroside (Fig. 7f,g). Furthermore, PHD3 overexpression diminished salidroside-induced VEGF-A, HO-1, PDGFB, HFG, FGF2, Nkb1, and ANG1 mRNA expression level in the ischemic hind limbs of HLI mice (Fig. 7h). Consistently, the effect of salidroside-induced VEGF-A, HO-1, PDGF-BB, HFG, FGF2 ands ANG1 protein expression, as well as HIF-1α protein accumulation were also abolished (Fig. 7i).

### Salidroside inhibits PHD3 through ERα/PHD3 axis

**Figure 8:**
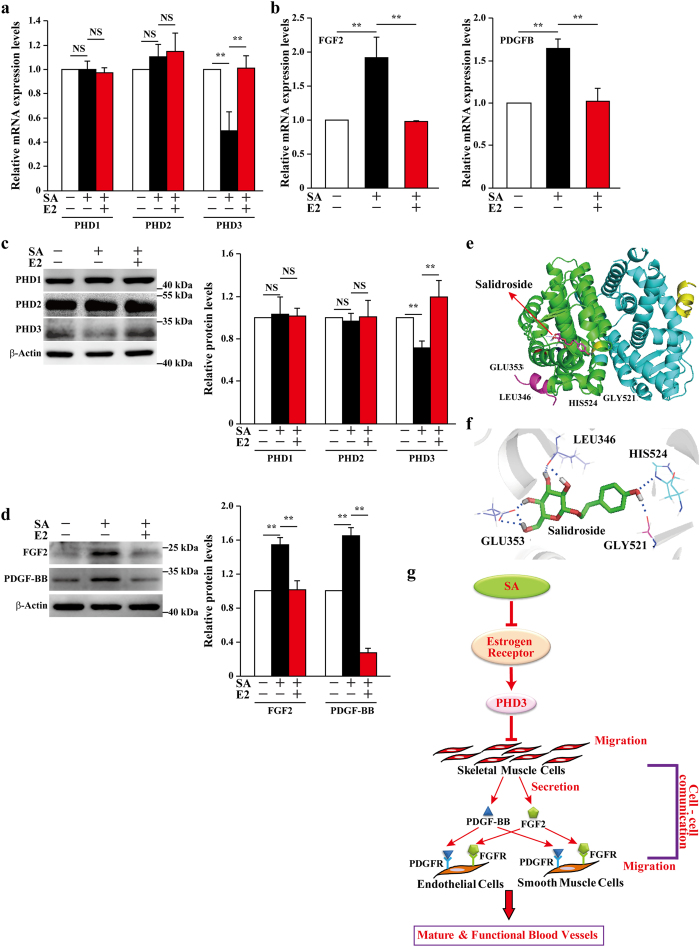
Salidroside exerts specific inhibitory effect on PHD3 via ERα/PHD3 axis. (**a**,**b**) The mRNA expression levels of PHDs family (**a**) and FGF2 and PDGFB (**b**) in the C2C12 cells treated with salidroside alone or salidroside and 17-β estradiol were analyzed by using quantitative RT-PCR (**p < 0.01). (**c**) The protein expression levels of PHDs family in the C2C12 cells treated with salidroside alone or salidroside and 17-β estradiol were analyzed by using western blotting: representative images (left) and quantitative analysis (right) (NS: not significant, **p < 0.01). (**d**) The protein expression levels of FGF2 and PDGF-BB in the C2C12 cells treated with salidroside alone or salidroside and 17-β estradiol were analyzed by using western blotting: representative images (left) and quantitative analysis (right) (**p < 0.01). (**e**,**f**) The model of salidroside-ERα binding predicted by using docking simulation: (**e**) zoom-out view of salidroside bound to ERα; (**f**) the predicted binding sites. Hydrogen bonds are presented as blue dashed lines. Interacting key residues are showen as line model. Salidroside was shown in stick representation with carbon and oxygen atoms depicted in blue and red, respectively. (**g**) Schematic diagram of the effect of salidroside on the neovascularization through ERα/PHD3 axis. All experiments were done in hypoxic condition. Quantitative data (**a**–**d**) were shown as means ± s.e.m of three independent experiments.

We next tried to reveal how salidroside affects the transcriptional level of PHD3. Very recent report showed that estrogen receptor alpha (ERα) could bind to PHD3 promoter, and upregulate PHD3 transcriptional activity^38^. Thus, we examined the possibility whether salidroside exerts its effects through ERα pathway. As shown in Fig. 8a, we did not observed any significant changes in the mRNA expression levels of PHD1 and PHD2 in C2C12 cells treated with salidroside irrespective of the presence of 17-β estradiol, an ERα agonist. However, the mRNA expression level of PHD3 was restored to the level of control upon 17-β estradiol addition (Fig. 8a). Furthermore, the increase of FGF2 and PDGFB mRNA expression levels were also suppressed when 17-β estradiol was added (Fig. 8b). Consistent with these, 17-β estradiol abolished salidroside-inhibition on the PHD3 protein level, as well as the upregulation effect of salidroside on the PHD3 downstream targets, *i.e.*, FGF2 and PDGF-BB (Fig. 8c,d). Moreover, we used the structure of ERα as reported in the PDB database (http://www.rcsb.org/pdb/home/home.do, structure ID: 4DMA) to conduct docking simulation. Using AutodockTools1.5.6 and Ledock 2013, we predicted the possibility of salidroside-ERα interaction, and found that salidroside might bind to ERα with a total binding energy of −5.5 kcal/mol as predicted by AutodockTools1.5.6 and −7.48 kcal/mol as predicted by Ledock 2013. The zoom-out view of salidroside bound to ERα and the predicted binding sites at LEU346, GLU353, GLY521 and HIS524 residues were shown in Fig. 8e,f, respectively. Together, these results showed the possibility salidroside might exerts its specific inhibitory effect on PHD3 through its interaction with ERα.

## Discussion

Although therapeutic angiogenesis had been considered as a potential strategy for treating HLI, effective and sufficient induction of mature and functional blood vessels remains challenging. To induce therapeutic angiogenesis, previous studies had demonstrated angiogenic factors-based strategy either in the form of protein or gene^2^. However, treatment with one or two angiogenic factors could not recapitulate neovascularization, as it is a complex process involving multiple angiogenic factors^2^. Furthermore, efficient delivery of macromolecules into target cells and their safety remains obstacles. Indeed, recent study has shown that despite of the promising early results, a phase III clinical trial using adenovirus vector encoding constitutively active HIF-1α failed to show benefit, and the lack of efficient gene transfer might be one of the reasons for this failure^39^. Our results firstly showed that localized, intramuscular administration of small-molecule drug salidroside could efficiently induced functional angiogenesis and blood perfusion recovery in HLI mice. As shown in the schematic diagram in Fig. 8g, salidroside, through its regulation on PHD3 transcriptional activity, promoted the mobility and paracrine signaling of skeletal muscle cells, which in turn activated skeletal muscle cells–endothelial and/or smooth muscle cells communication, promoted endothelial and smooth muscle cells mobility, and subsequently improved neovascularization in HLI mice. Moreover, although systemic toxicity and side effects need to be studied more thoroughly, *Rhodiola* itself has been used as a herbal medicine for centuries, and in consistent with this, our preliminary results did not reveal any obvious morphological side effects in liver, kidney, spleen and heart upon prolonged administration of salidroside. To our knowledge, our study is the first one which showed that small molecule drug targetting PHD3 transcription could exert effective therapeutic angiogenesis in HLI.

PHD family members, which play central roles in the cellular response to hypoxia, has been shown as crucial regulators in neovascularization. Surprisingly, despite of their important roles, simultaneous deletion of multiple PHD genes not only failed to display improved vascular and muscular integrity but also caused marked hepatotoxicity and conspicuous skin redness, indicating the importance of a PHD-specific inhibitor in therapeutic angiogenesis^14^,^36^,^40^,^41^,^42^,^43^. However, due to their high similarity in protein conformation, especially in the highly conserved 2-OG binding pocket domain, current small molecule inhibitors hardly show PHD-isoform selectivity^44^,^45^. A recent report identified an estrogen response element (ERE) in the proximal promoter region of PHD3, and showed that ERα upregulates PHD3 transcriptional activity in adipose tissue^38^. These facts, together with our results which showed that salidroside negatively regulated the transcriptional levels of PHD3, prompted the possibility that salidroside exerts its PHD3 inhibitory effect via ERα regulatory pathway. Indeed, our results showed that in skeletal muscle cells, addition of ERα agonist, 17-β estradiol, abolished the specific inhibitory effect of salidroside on PHD3, and consequently, the inductions of angiogenic factors. Furthermore, by using two different molecular docking simulations, we found that salidroside could bind to ERα, plaucibly through its LEU346, GLU353, GLY521 and HIS524 residues. Although more detail investigations, including crystallography analysis and point mutations of the predicted binding sites need to be conducted to confirm their bindings, our data indicated, for the first time, the potential cellular binding partner of small-molecule drug salidroside.

PHD3 was firstly identified as a prolyl-hydroxilase responsible for regulating HIF-1 stability in an oxygen-dependent manner^46^. It is thought to be the primary regulator of HIF-1 under severe and prolonged hypoxia as it showed the most robust induction under hypoxia, due to the presence of a feedback mechanism^35^,^36^,^47^,^48^,^49^,^50^,^51^,^52^,^53^. On the other hand, skeletal muscle has been known to play important roles in neoangiogenesis because of its ability to secrete various soluble angiogenic factors, and thus, stimulating the expression and secretion of skeletal muscle angiogenic factors might be a key to successful therapeutic angiogenesis^10^,^12^,^13^,^14^,^31^. In this study, we uncovered a novel function of PHD3 in regulating skeletal muscle cells paracrine signaling. Furthermore, we showed that PHD3 inhibition significantly increased the amount of angiogenic factors secreted from skeletal muscle cells, and that PHD3 inhibition enhance cross-talk between skeletal muscle cells and endothelial and/or smooth muscle cells via paracrine signals especially through FGF2/FGF2R and PDGF-BB/PDGFR-β pathways. In addition, our study also showed that PHD3 inhibition could enhanced F-Actin polymerization and the migration potential of skeletal muscle cells, which is consistent with previous study showing that PHD3 mediated prolyl hydroxylation of nonmuscle actin, and impaired actin polymerization in cervical cancer cells^54^. Endothelial and smooth muscle cells, which form mature blood vessels, have been reported to extend their pseudopodia toward the angiogenic stimuli^29^, thus, although further detailed study is needed to elucidate the role of smooth muscle cells migration potential in inducing therapeutic angiogenesis, we reasoned that the enhanced mobility of skeletal muscle cells might improve the dissemination of the skeletal muscle cells-secreted angiogenic factors to endothelial and smooth muscle cells, and resulted in the induction of angiogenesis in a larger area.

Angiogenesis is a complex process involving a number of angiogenic factors. Our study demonstrated that salidroside, through inhibiting PHD3, could activates the expression of multiple angiogenic factors from the skeletal muscle cells, including VEGF, HGF, PDGF-BB, FGF2 and ANG1, all of them play critical roles in forming mature and functional blood vessels. Here we focused on the role of PDGF-BB and FGF2 in the salidroside-induced neovascularization, as salidroside could induce the migration of both endothelial and smooth muscle cells. PDGF-BB had been known to be secreted by endothelial cells to recruit pericytes^33^,^34^. Furthermore, as shown by our results (Supplementary Fig. 11a,b for immunohistochemistry staining against PDGF-BB and skeletal muscle cells marker myoglobin, and against PDGF receptor beta (PDGFR-β) and PECAM-1, respectively) and other groups previously, PDGF-BB is also expressed by skeletal muscle cells^15^, while its receptor could be expressed on endothelial cells^27^,^34^. Moreover, Chiu *et al*. demonstrated that PDGF-BB/PDGFR-β pathway could induce endothelial cells migration^32^. It has also been reported that FGF2 and PDGF-BB showed synergistic positive effects on angiogenesis, probably due to the upregulation of PDGFR-β expression by FGF2, and the increase of smooth muscle cell responsiveness to FGF2 upon treatment with PDGF-BB^16^,^30^. Indeed, our results conformed with these previous reports, as silencing both the ligand and the receptor of FGF2/FGF2R and PDGF-BB/PDGFR-β pathways could significantly suppressed endothelial and smooth muscle cells mobility. Furthermore, suppression of either the ligands or the receptors of these pathways robustly reduced the positive effect of salidroside on endothelial and smooth muscle cells mobility. Thus, although it is most plausibly that the effect of salidroside on neovascularization is the results of synergistic cooperation between the angiogenic factors secreted from the skeletal muscle cells, our results demonstrated that FGF2/FGF2R and PDGF-BB/PDGFR-β axis play important roles in activating cell–cell communication between skeletal muscle cells and endothelial and/or smooth muscle cells during this process. Nevertheless, to fully reveal the molecular mechanism of salidroside-induced neovascularization, it is important to elucidate the role of other angiogenic factors in future studies.

In conclusion, our study presents a novel drug-based investigation using salidroside for the therapeutic angiogenesis in HLI. Our *in vitro* and *in vivo* results concomitantly showed that salidroside is a PHD3 specific inhibitor that suppresses PHD3 transcription via ERα-regulated pathway, and exerts significant enhancement in the formation of mature blood vessels and blood perfusion recovery in HLI mice. Furthermore, to our knowledge, our findings is the first one that revealed molecular mechanism of salidroside on modulating gene expression, and thus might provide new insights into the mechanism and functions of salidroside. Moreover, our novel findings also showed that PHD3 is involved in the crosstalk between skeletal muscle cells and endothelial and/or smooth muscle cells, suggesting that PHD3 acts as a crucial paracrine signalling regulator in vascular remodelling. Thus, our study proposed that targetting skeletal muscle cells ERα/PHD3 axis using salidroside might be a potential small molecule-based therapeutic strategy for HLI and other ischemic disease.

## Methods

### Cell culture and cell experiments

The C2C12, MOVAS, and HUVEC cell lines were obtained from the American Type Culture Collection (ATCC). C2C12 and MOVAS cells were maintained in DMEM (Gibco, Grand Island, NY) supplemented with 10% fetal bovine serum (Biological Industries, Israel). HUVECs were cultured in an EGM-2 (endothelial growth medium-2) supplemented with growth factors and antibiotics (Lonza, Walkersville, MD). Cells were cultured in 37 °C. The cell lines had been tested periodically for mycoplasma contamination by using Mycoplasma Detection Kit-QuickTest (Biotool, Houston, TX).

For the overexpression experiment, cells were cultured in a 6-well plate and transfected with 2 μg pcPHD3 or pcDNA3.1(+) by using Lipofectamine 2000 (Invitrogen, Carlsbad, CA) according to the manufacturer’s instructions, washed with PBS 24 h later and treated with salidroside (purity ≥98%, Tauto Biotech, Shanghai, China, final concentration: 100 μg/ml).

For the gene silencing experiment, cells were cultured in a 6-well plate and transfected with control vector (shCon) or shRNA expression vectors targeting specific genes by using Lipofectamine 2000 (Invitrogen) according to the manufacturer’s instruction. Twenty-four hours after transfection, the cells were treated with puromycin (final concentration: 2.5 μg/ml) for 48 h to eliminate untransfected cells.

For hypoxia experiments, cells were incubated in a hypoxia chamber (Anaeropouch Box, 0.1% O_2_, Mitsubishi GAS Chemical, Tokyo, Japan) for 6 h or 12 h before RNA or protein extraction, respectively.

For cells subjected to salidroside treatment, the cells were treated with salidroside (final concentration: 100 μg/ml) for 24 h before exposure to hypoxia. For cells transfected with shRNA or overexpression plasmid, the total RNA and protein were extracted after incubation in the hypoxia chamber for 12 h and 24 h, respectively.

For experiments using 17-β estradiol, the cells were treated with salidroside (100 μg/ml) and 17-β estradiol (Alladin, Shanghai, China, final concentration of 10 nM) for 24 h, washed and further cultured with 17-β estradiol under hypoxic condition for 6 h or 12 h for RNA and protein experiment, respectively.

### Plasmids and constructs

To construct murine PHD3 (NM_028133.2), FGF2 (NM_008006.2), PDGFB (NM_011057.3), FGF2R (NM_010206.3) and PDGFR-β (NM_001146268.1) shRNA expression vectors, specific target sites for murine PHD3, FGF2, PDGFB, FGF2R and PDGFR-β were predicted using the algorithm for predicting specific and high-efficiency RNAi target sites: shPHD3–1 (AGA TAT TTC TCT TTC TTG C) and shPHD3–2 (TTG TTA TGG ACG ATG AAC C); shFGF2–1 (GCA AGA ACG GCG GCT TCT T) and shFGF2–2 (GCG AGA AGA GCG ACC CAC A); shPDGFB-1 (GGT CCA GGT GAG AAA GAT T) and shPDGFB-2 (AAG TCG CTC TTC TTG GTG C); shFGF2R-1 (AAG AGG AGG CAC TTC CAG C) and shFGF2R-2 (GCT GGG AGT GTC TCT TCT T); shPDGFR-β-1 (TAT AGA TTC ATC CTT GCT C) and shPDGFR-β-2 (TGG AGA TGT AGT TTG AGT C); then oligonucleotides with a hairpin structure, overhanging sequences, and a terminator were synthesized, annealed, and inserted into the *Bsp*MI sites of a pcPUR + U6i cassette vector containing the U6 promoter as described previously^55^,^56^. As a control, we used a vector containing a stretch of 7 thymines immediately downstream of the U6 promoter (shCon).

For the PHD3 overexpression vector, murine cDNA was obtained by reverse-transcribing total RNA extracted from C2C12 myoblast cells using the PrimeScript RT Reagent Kit with gDNA Eraser (Takara Bio), then the coding region of murine PHD3 was amplified using the Takara Ex Taq Kit (Takara Bio, Dalian, China). The amplicon was inserted into the pMD19T plasmid (Takara Bio) and subcloned into the *Bam*HI and *Not*I sites of pcDNA3.1(+) (Invitrogen).

All plasmids were purified using Endo-free plasmid Maxi Kit (Qiagen, Hilden, Germany).

### Experimental animals

Male Balb/c mice (inbred strain, 8 weeks old) were purchased from the Third Military Medical University (Chongqing, China). Animal studies were carried out in the Third Military Medical University (Chongqing, China, Permit Number SYXK-PLA-20120031), and approved by the Laboratory Animal Welfare and Ethics Committee of the Third Military Medical University. All surgeries were performed under ketamine/xylazine anesthesia (intraperitoneal administration, 80 mg/kg body weight and 50 mg/kg body weight, respectively), and all efforts were made to minimize suffering.

### Hind-limb ischemia model

Eight-week-old male Balb/c mice were anesthetized as described above. The leg was shaved and depilated, and the proximal part of the left femoral artery was completely excised to obtain the hind-limb ischemia mouse model^14^,^57^,^58^. The right femoral artery was not excised and used as a control. Salidroside (100 mg/kg body weight dissolved in PBS) or PBS was administered intramuscularly into the gastrocnemius muscle one day after femoral artery excision and every 3 days thereafter (Supplementary Fig. 1a). For FGF2 and PDGF pathway inhibition experiments, FGF2R inhibitor (PD173074, 1 mg/kg body weight), PDGFR inhibitor (CP868596, 0.4 mg/kg body weight), both of them dissolved in solvent (30% PEG400, 0.5% Tween-80, and 5% propylene glycol dissolved in saline (0.9% NaCl)), or solvent was administered intramuscularly into the gastrocnemius muscle immediately after femoral artery excision and every 2 days thereafter (Supplementary Fig. 1b). For the PHD3 overexpression experiment, 20 μg pcPHD3 or pcDNA3.1(+) plasmid dissolved in 100 μl PBS was injected intramuscularly once a week starting from the day after surgery (Supplementary Fig. 1c). Ischemic damage was visually assessed and scored as reported previously (0 = no difference from the non-ischemic hind limb, 1 = mild discoloration, 2 = moderate discoloration, 3 = severe discoloration, subcutaneous tissue loss, or necrosis, and 4 = amputation) at indicated time points^58^. The mice were allocated randomly after surgery, and the investigator was blinded to the group allocation and during the assessment. Mice that experience amputation were euthanized to minimize their suffering according to the Laboratory Animal Welfare and Ethics Committee of the Third Military Medical University.

### Laser Doppler Perfusion Imaging

Mice were anesthetized as described above and blood perfusion in both ischemic (left) and non-ischemic hind limbs (right) was measured using Laser Doppler Perfusion Imaging (LDPI) (Moor Instruments Ltd, Axminster, Devon, England) before surgery, directly after surgery, and on days 3, 7, 14, and 21 after surgery. Blood perfusion ratio was obtained by normalizing the blood perfusion of ischemic hind limbs with that of the contralateral non-ischemic hind limbs.

### Preparation of conditioned medium

C2C12 cells were treated with salidroside (final concentration: 100 μg/ml) for 24 h, washed, and incubated under hypoxia for 24 h. The culture medium was collected and filtrated through a 0.22-μm filter to obtain conditioned medium (CM-SA). For controls, cells were treated with PBS instead of salidroside for 24 h and cultured under normoxic or hypoxic condition for 24 h, and conditioned media (CM-N and CM-H, respectively) were collected as described above.

Conditioned media from C2C12 cells transfected with shFGF2 plasmid, shPDGFB plasmid or both of them or shCon plasmid and treated with salidroside (CM-shFGF2/SA, CM-shPDGFB/SA, CM-shFGF2-shPDGFB/SA and CM-shCon/SA, respectively) or PBS (CM-shFGF2-H, CM-shPDGFB-H and CM-shCon-H) were collected as described above. Conditioned medium from C2C12 cells transfected control plasmid and treated with PBS for 24 h and then cultured under normoxic condition for 24 h (CM-shCon-H) were also collected as described above.

Conditioned media from C2C12 cells transfected with PHD3 overexpression plasmid and treated with salidroside (CM-pcPHD3/SA) or transfected with control plasmid and treated with salidroside (CM-pcDNA/SA) or PBS (CM-pcDNA) were prepared as follows: C2C12 cells were transfected with pcPHD3 or pcDNA3.1(+) plasmid for 24 h, treated with 100 μg/ml salidroside or PBS, washed, exposed to hypoxia for 24 h, and conditioned media were collected as described above.

### Protein array

The Proteome Profiler Mouse Angiogenesis Antibody array (R&D Systems, Minneapolis, MN) was used according to the manufacturer’s protocol. In brief, nitrocellulose membranes spotted with antibodies against different angiogenesis-related proteins were first incubated in blocking buffer for 1 h. Medium from C2C12 cells treated with salidroside (final concentration: 100 μg/ml) or PBS were prepared, equal amounts of total protein (350 μg) from each sample were dissolved in 500 μl DMEM, and mixed with a cocktail of biotin-labeled antibodies against different angiogenesis-related proteins and applied to the membranes which were incubated at 4 °C overnight. The membranes were then washed and incubated with horseradish peroxidase (HRP) conjugated streptavidin for 1 hour at room temperature, and the signals were detected according to the manufacturer’s protocol. Semiquantitative analysis was performed by using Image Lab (Bio-Rad, Hercules, CA).

### Flow Cytometry

Cells were treated with PBS or salidroside (final concentration: 100 μg/ml) and incubated under normoxic condition for 24 h, washed and incubated under hypoxic condition for 24 h, then the number of apoptotic cells were analyzed by using Annexin V-FITC/PI Apoptosis Detection Kit (KeyGen Biotech, Jiangsu, China) according to the manufacturer’s instruction. Briefly, the cells were trypsinized and re-suspended in binding buffer containing Annexin V-FITC and PI at room temperature for 10 min before being analyzed by using FACS Calibur (BD Biosciences). For experiments with conditioned medium, cells were cultured with conditioned medium under hypoxia for 24 h before the number of apoptotic cells was analyzed.

### Docking simulation

The crystal structure of ERα, as had been reported previously^59^, was obtained from the PDB database (http://www.rcsb.org/pdb/home/home.do, ID: 4DMA). AutodockTools1.5.6 (Scripps Research Institute) and Ledock 2013 (Lephar Research)^60^,^61^ were used to predict the putative interaction between ERα and salidroside. Standard cleaned up procedure was employed where salidroside was checked for valence errors, salt and solvent removal. The binding pocket residues were not allowed to be flexible, but the ligand was allowed to be flexible. Only the best ligand-binding energy predicted from the two simulation systems were showed. The 3D model for ERα was built using PyMOL 1.8 X.

### Statistical analysis

The quantitative data shown in this study were expressed as the means ± s.e.m from three independent experiments. Statistical analysis were performed using SPSS Statistics v. 17.0 software. Τhe mouse experimental data were analyzed using a nonparametric Mann-Whitney test. Statistical significance of the differences between two groups was analyzed by Student’s *t*-test at a signification level of *p < 0.05 and **p < 0.01.

## Additional Information

**How to cite this article**: Zhang, J. *et al*. Inhibition of PHD3 by salidroside promotes neovascularization through cell–cell communications mediated by muscle-secreted angiogenic factors. *Sci. Rep.* **7**, 43935; doi: 10.1038/srep43935 (2017).

**Publisher's note:** Springer Nature remains neutral with regard to jurisdictional claims in published maps and institutional affiliations.

## Supplementary Material

Supplementary Information
