# Supplementary material for: Inhibition of PHD3 by salidroside promotes neovascularization through cell–cell communications mediated by muscle-secreted angiogenic factors
Source: Sci Rep. 2017 Mar 7;7:43935. doi: 10.1038/srep43935 (PMC5339704; doi:10.1038/srep43935)
Supplement: Supplementary Information [file srep43935-s1.pdf]

Supplementary Information for

"Inhibition of PHD3 by salidroside promotes neovascularization through cell-cell communications mediated by muscle-secreted angiogenic factors"

Jing Zhang[1], Vivi Kasim[1,*], Yu-dan Xie[1], Can Huang[1], Julita Sisjayawan[1], Agnes Dwi Arianti[1], Xue-song Yan [1], Cai-ping Liu[1], Xiao-yan Wu[1], Li Yang[1,2], Makoto Miyagishi[3], Shou-rong Wu[1,2,*]

[1]The Key Laboratory of Biorheological Science and Technology, Ministry of Education, College of Bioengineering, Chongqing University, Chongqing 400044, China.

[2]The 111 Project Laboratory of Biomechanics and Tissue Repair, College of Bioengineering, Chongqing University, Chongqing 400044, China.

[3]Molecular Composite Medicine Research Group, Biomedical Research Institute, National Institute of Advanced Industrial Science and Technology (AIST), Tsukuba 305-8566, Japan.

* Corresponding authors:

Shou-Rong Wu, Ph.D.

The Key Laboratory of Biorheological Science and Technology, Ministry of Education, Chongqing University, 174 Shazheng Street, Shapingba, Chongqing 400044, China

Phone: +86-23-65111632; Fax: +86-23-65111802;

E-mail: shourongwu@cqu.edu.cn

Vivi Kasim, Ph.D.

The Key Laboratory of Biorheological Science and Technology, Ministry of Education, Chongqing University, 174 Shazheng Street, Shapingba, Chongqing 400044, China

Phone: +86-23-65112672; Fax: +86-23-65111802;

E-mail: vivikasim@cqu.edu.cn

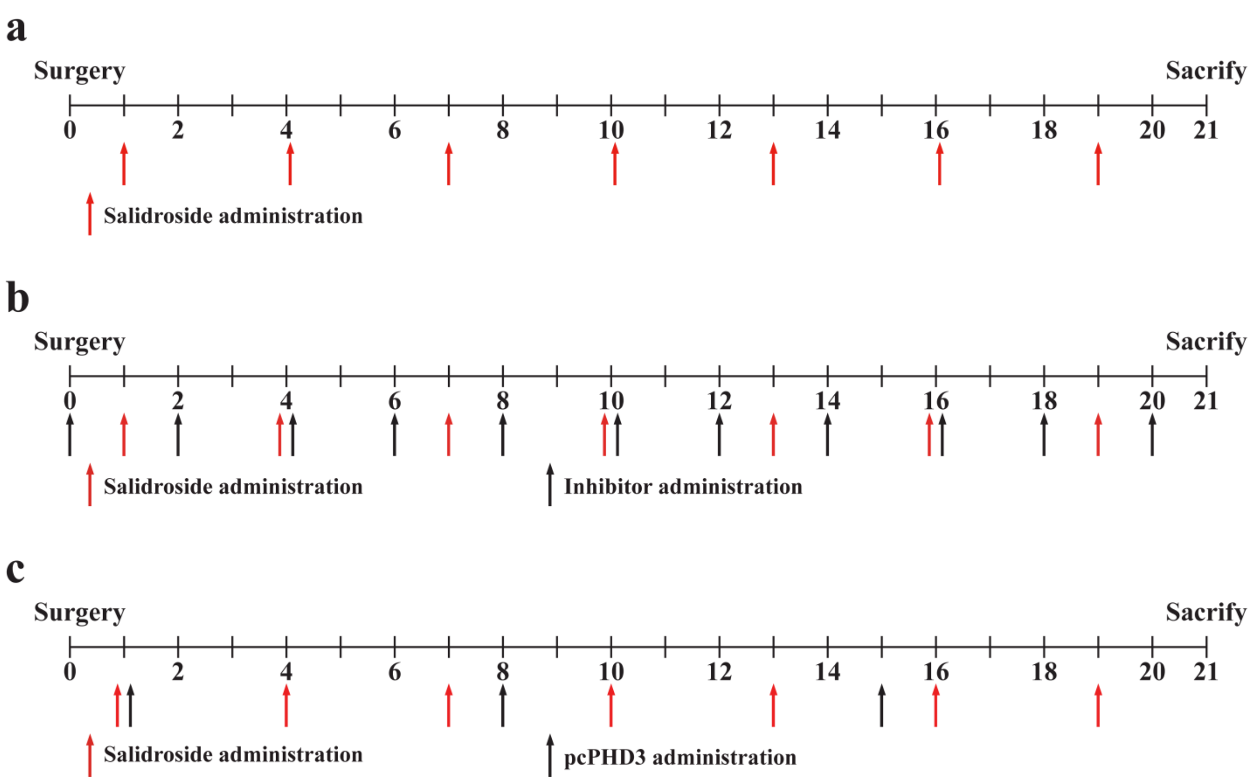

Supplementary Figure 1. Schematic diagram for the administration schedule of salidroside. (**a**) Salidroside was administered intramuscularly into the gastrocnemius muscle of the ischemic hind limb one day after femoral artery excision and every three days thereafter. (**b**) Salidroside was administered intramuscularly into the gastrocnemius muscle of the ischemic hind limb one day after femoral artery excision and every three days thereafter; while FGF2R inhibitor (PD173074) or PDGFR inhibitor (CP868596) was injected intramuscularly into the gastrocnemius muscle of the ischemic hind limb right after femoral artery excision and every two days thereafter. (**c**) Salidroside was administered intramuscularly into the gastrocnemius muscle of the ischemic hind limb one day after femoral artery excision and every three days thereafter; while PHD3 overexpression plasmid were injected intramuscularly into the gastrocnemius muscle of the ischemic hind limb once a week starting from the day after femoral artery excision.

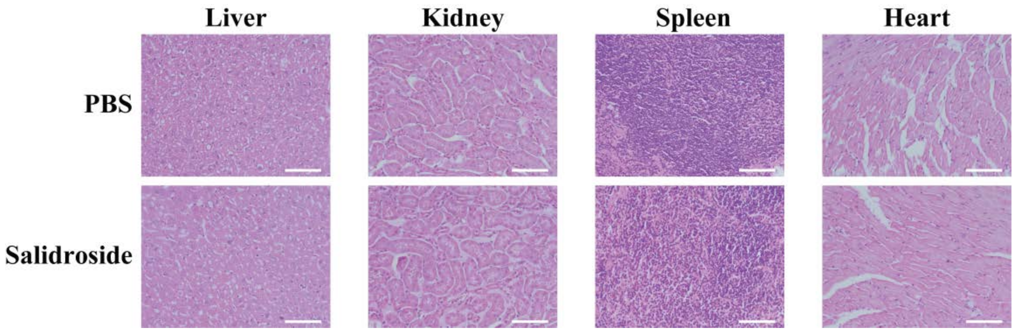

Supplementary Figure 2. Prolonged salidroside administration does not result in obvious morphological changes. Salidroside was administered intramuscularly into the gastrocnemius muscle of the ischemic hind limb of HLI mice every three days for two months starting from the day after femoral artery excision. Representative Hematoxylin & Eosin stainings of liver, kidney, spleen and heart were shown. Scale bars: 100 μm.

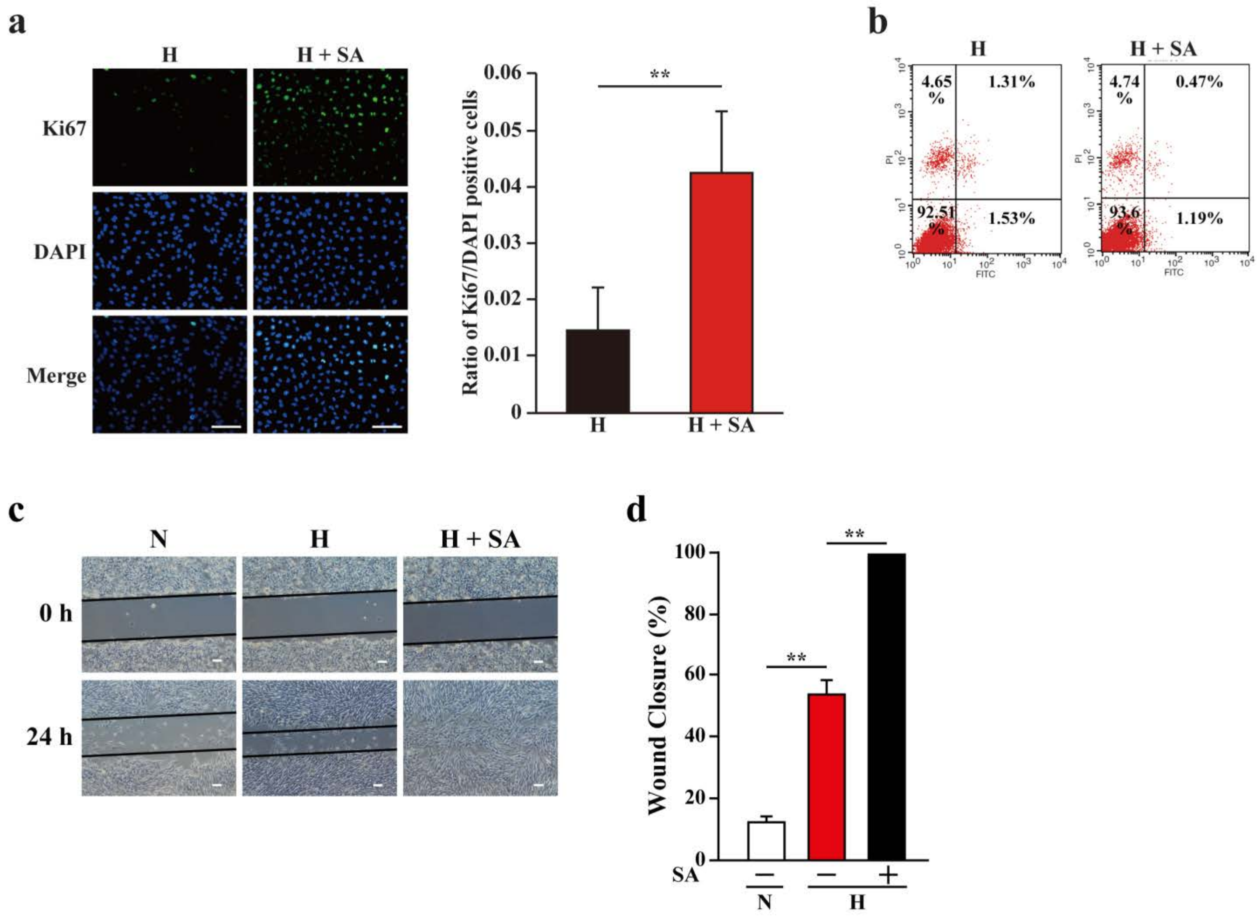

Supplementary Figure 3. Salidroside promotes skeletal muscle cells proliferation and migration. (**a**) The proliferation of C2C12 cells treated with salidroside or PBS and cultured in hypoxia was analyzed using Ki67 staining. The nuclei were stained with DAPI. The representative microscopic images (Scale bars: 100 μm）were shown in left side; ratio of Ki67 positive cells to DAPI positive cells was shown in right side (**p < 0.01). (**b**) The percentage of apoptotic cells in C2C12 cells treated with salidroside or PBS and cultured in hypoxia were analyzed by using Annexin V-FITC/PI staining and FACS analysis. **(c,d)** the mobility of C2C12 cells treated with salidroside and cultured in hypoxia or treated with PBS and cultured in hypoxia or normoxia were analyzed using scratch assay: **(c)** representative images (scale bars: 100 μm) and **(d)** percentage of wound closure (**p < 0.01). N: normoxia, H: hypoxia, SA: salidroside. The quantitative results (**a** and **d**) are the means ± s.e.m of three independent experiments.

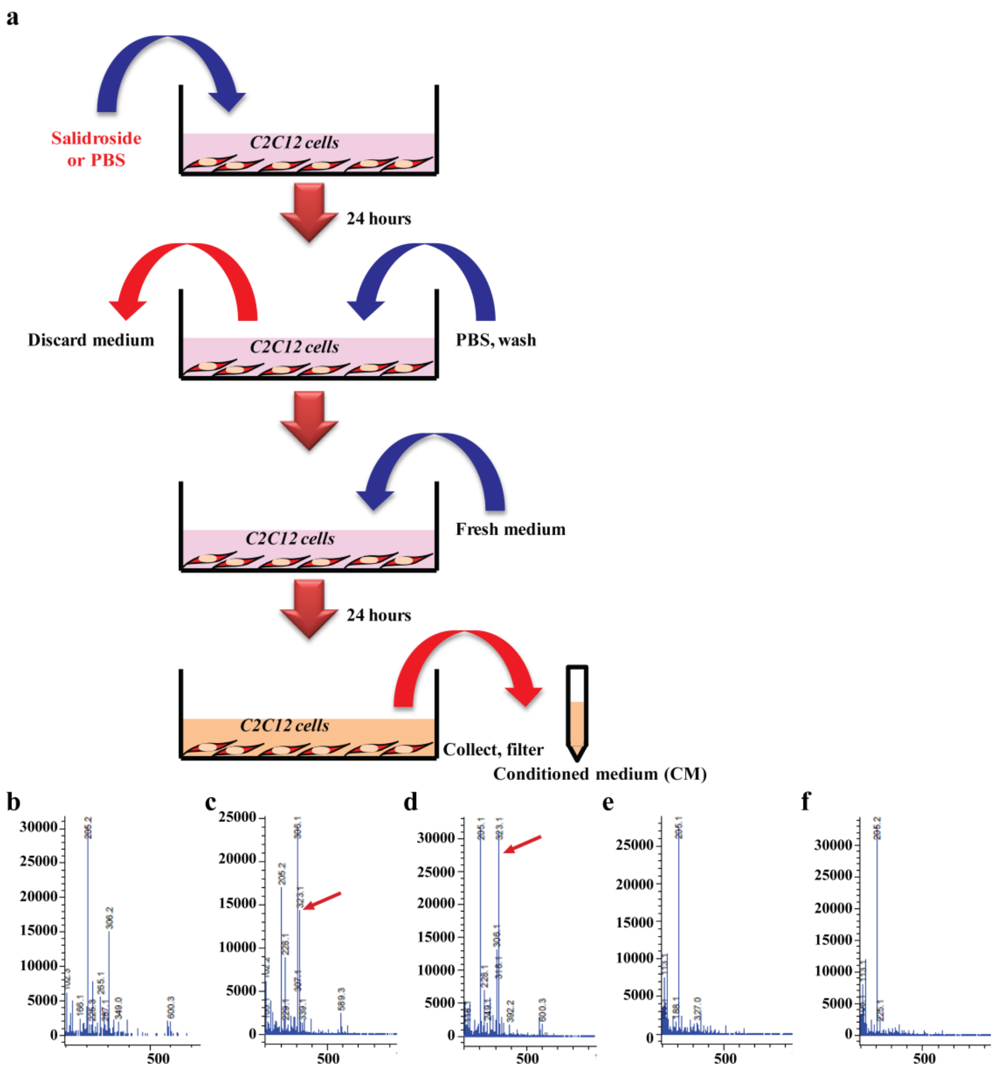

Supplementary Figure 4. Schematic diagram of the preparation and HPLC-MS analysis of conditioned medium collected from salidroside-treated C2C12 cells. (a) Salidroside (final concentration 100 µg/ml) or PBS was added into the culture medium of C2C12 cells, and twenty-four hours later, the medium was discarded, then the cells were washed twice with PBS. After the addition of fresh medium, cells were further cultured for 24 h in normoxia or hypoxia, and then the medium was collected and filtered using a 0.22-µm filter. **(b-f)** HPLC-MS analysis results: **(b)** DMEM supplemented with 10% FBS; **(c)** standard reference of salidroside; **(d)** culture medium collected from C2C12 cells cultured with DMEM supplemented with 10% FBS and salidroside for 24 h in normoxia; **(e)** culture medium collected from C2C12 cells cultured with DMEM supplemented with 10% FBS and salidroside for 24 h in normoxia, washed with PBS twice, added with fresh DMEM supplemented with 10% FBS and cultured in hypoxia for 0 h; **(f)** culture medium collected from C2C12 cells cultured with DMEM supplemented with 10% FBS and salidroside for 24 h in normoxia, washed with PBS twice, added with fresh DMEM supplemented with 10% FBS and cultured in hypoxia for 24 h (*i.e.*, CM-SA). Salidroside's peak, as indicated by red arrow, could be detected at 323.1 U.

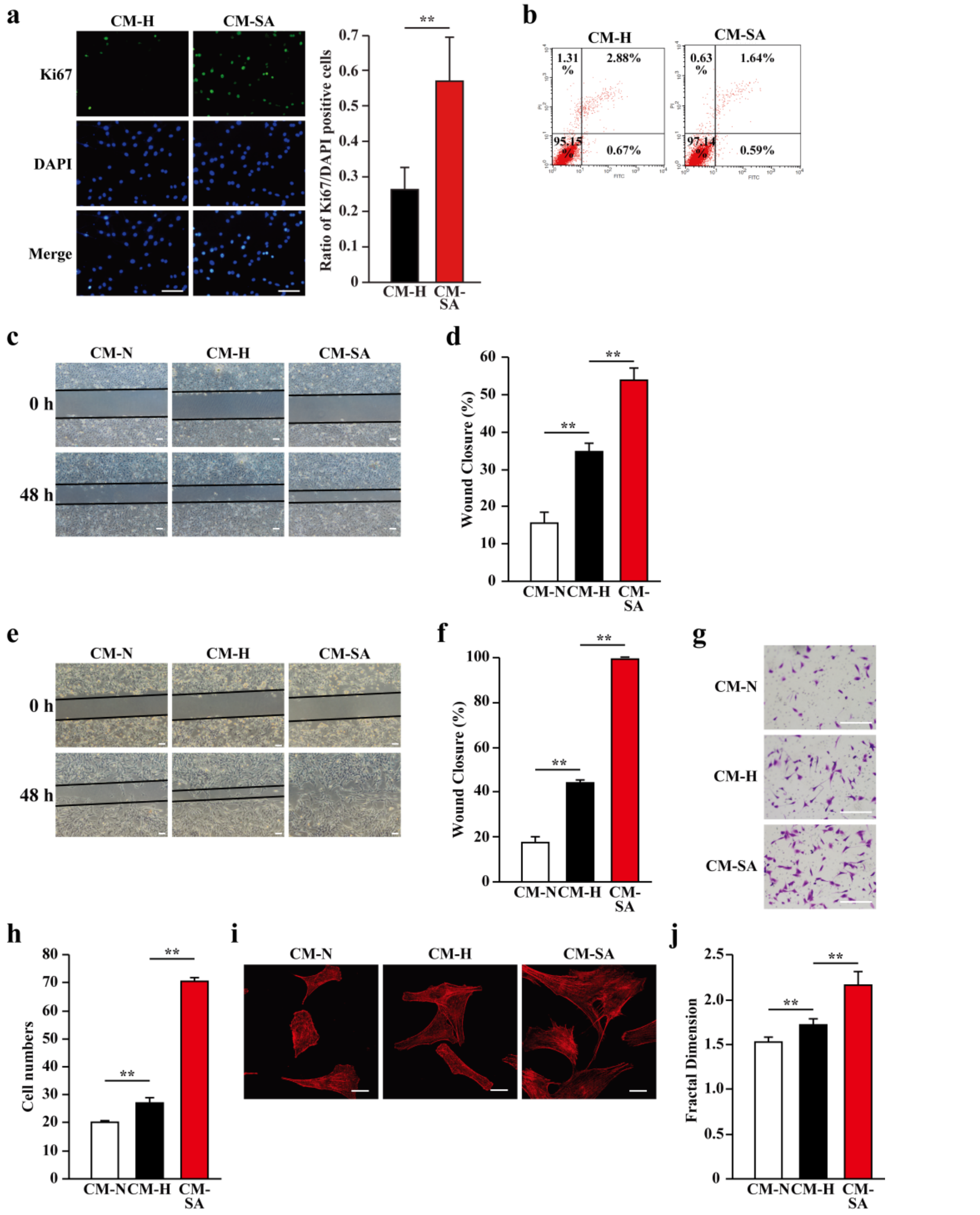

Supplementary Figure 5. Salidroside-induced skeletal muscle cells secretome enhances the proliferation and mobility of endothelial and smooth muscle cells. (**a**) The proliferation of MOVAS cells cultured with CM-SA were analyzed using Ki67 staining. The nuclei were stained

with DAPI. Left: representative images; right: ratio of Ki67 positive cells to DAPI positive cells (**$p < 0.01$). (**b**) The percentage of apoptotic cells in MOVAS cells cultured with CM-SA were analyzed using Annexin V-FITC/PI staining and FACS analysis. (**c, d**) The mobility of HUVECs cultured with CM-SA were examined using scratch assay; **(c)** representative images and (**d**) percentage of wound closure (**$p < 0.01$). (**e-h**) The mobility of MOVAS cells cultured with CM-SA were examined by using scratch assay: **(e)** representative images and (**f**) percentage of wound closure (**$p < 0.01$); and transwell chamber assay: (**g**) representative images and (**h**) quantification of migrated cells (**$p < 0.01$). (**i, j**) Morphological changes of F-actin were examined using phalloidin staining: **(i)** representative images; **(j)** the quantification analysis of fractal dimension (**$p < 0.01$). CM-SA: conditioned medium from salidroside-treated C2C12 cells cultured under hypoxia; CM-N and CM-H: conditioned medium from PBS-treated C2C12 cells cultured under normoxia or hypoxia, respectively. Scale bars: 100 μm (scratch and transwell chamber assays) or 25 μm (phalloidin staining). All experiments were done in hypoxic condition. The quantitative results (**a, d, f, h and j**) are the means ± s.e.m of three independent experiments.

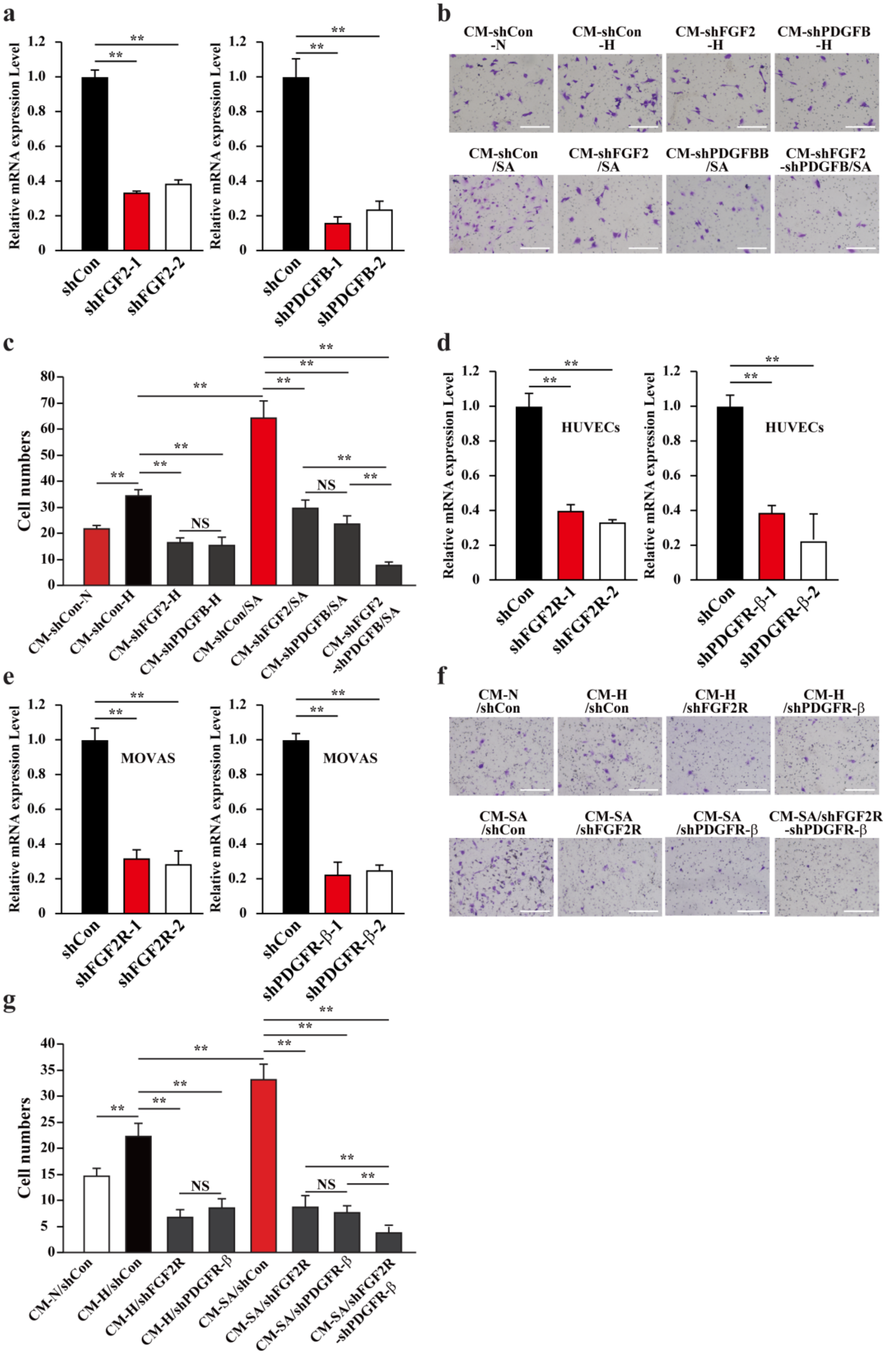

Supplementary Figure 6. Salidroside mediates skeletal muscle–endothelial and/or smooth muscle cells communications by PDGF-BB/PDGFR-β and FGF2/FGF2R axis. (**a**) The expression levels of FGF2 and PDGFB in C2C12 cells transfected with shCon, shFGF2-1,

shFGF2-2, shPDGFB-1 or shPDGFB-2 and cultured in hypoxic condition were analyzed by using quantitative RT-PCR (**p < 0.01, data were shown as relative to the expression level in the cells transfected with shCon). (**b, c**) The mobility of MOVAS cells cultured with CM-shFGF2/SA, CM-shPDGFB/SA or CM-shFGF2-shPDGFB/SA were analyzed by using transwell chamber assay: (**b**) representative images and (**c**) quantification of migrated cells (NS: not significant, **p < 0.01). (**d,e**) The expression levels of FGF2R and PDGFR-β in HUVECs (**d**) and MOVAS (**e**) cells transfected with shCon, shFGF2R-1, shFGF2R-2, shPDGFR-β-1 or shPDGFR-β-2 and cultured in hypoxic condition were analyzed by using quantitative RT-PCR (**p < 0.01, data were shown as relative to the expression level in the cells transfected with shCon). (**f, g**) The mobility of FGF2R- or PDGFR-β-silenced MOVAS cells cultured with CM-SA were analyzed by using transwell chamber assay: (**f**) representative images and (**g**) quantification of migrated cells (NS: not significant, **p < 0.01). CM-shCon-N and CM-shCon-H: conditioned medium from shCon-transfected, PBS-treated C2C12 cells cultured under normoxia or hypoxia, respectively; CM-FGF2-H and CM-shPDGFB-H: conditioned medium from FGF2- or PDGFB-silenced C2C12 cells cultured under hypoxia, respectively; CM-shCon/SA, CM-shFGF2/SA, CM-shPDGFB/SA and CM-shFGF2-shPDGFB/SA: conditioned medium from shCon-transfected, FGF2-silenced, PDGFB-silenced or FGF2- and PDGFB-silenced, salidroside-treated C2C12 cells cultured under hypoxia; CM-N/shCon, CM-H/shCon, CM-H/shFGF2R, CM-H/shPDGFR-β, CM-SA/shCon, CM-SA/shFGF2R, CM-SA/shPDGFR-β and CM-SA/shPDGFR-β-shFGF2R: MOVAS transfected with shCon, shFGF2R or shPDGFR-β and cultured with CM-N, CM-H or CM-SA. Scale bars: 100 μm (transwell chamber assays). All experiments were done in hypoxic condition. The quantitative results (**a, c, d, e and g**) are the means ± s.e.m of three independent experiments.

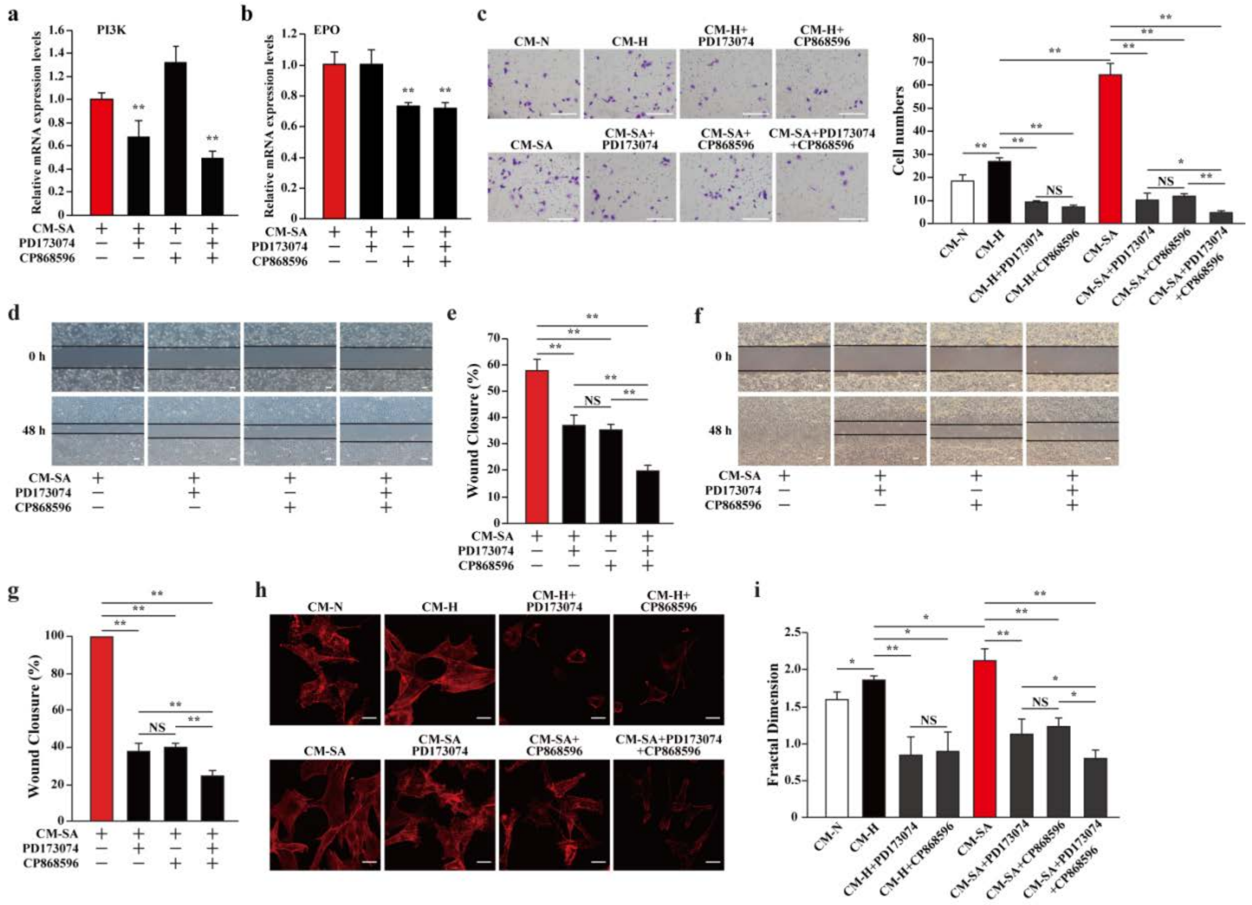

Supplementary Figure 7. Blocking of PDGFR-β and FGF2R abrogated salidroside-mediated skeletal muscle–endothelial and/or smooth muscle cells communication. (a,b) The expression levels of: (**a**) FGF2/FGF2R downstream gene PI3K; and (**b**) PDGF-BB/PDGFR-β downstream gene EPO in MOVAS cells treated with CM-SA and with PD173074 (20 nM), CP868596 (0.3 μM) or both of them were analyzed by using quantitative RT-PCR (**p < 0.01, data were shown as relative to the expression level in the cells treated with CM-SA only). (**c**) The mobility of MOVAS cells cultured with CM-SA and PD173074, CP868596 or both of them were analyzed by using transwell chamber assay: representative images (left) and quantification of migrated cells (right) (NS: not significant, **p < 0.01). (**d-g**) The mobility of HUVECs and MOVAS cells cultured with CM-SA and PD173074, CP868596 or both of them were analyzed by using scratch assay: (**d**) representative images and (**e**) percentage of wound closure (NS: not significant, **p < 0.01) for HUVECs; (**f**) representative images and (**j**) percentage of wound closure (**p < 0.01) for MOVAS. (**h,i**) Morphological changes of F-actin were examined by using phalloidin staining: **(h)** representative images; **(i)** fractal dimension analysis (NS: not significant, *p < 0.05, **p < 0.01). The final concentration for PD173074 and CP868596 were 20 nM and 0.3 μM, respectively. SA: salidroside; CM-N and CM-H: conditioned medium from PBS-treated C2C12 cells cultured under normoxia or hypoxia, respectively; CM-SA: conditioned medium from salidroside-treated C2C12 cells cultured under hypoxia. Scale bars: 100 μm (scratch and transwell chamber assays) or 25 μm (phalloidin

staining). All experiments were done in hypoxic condition. The quantitative results (**a, b, c, e, g and i**) are the means ± s.e.m of three independent experiments.

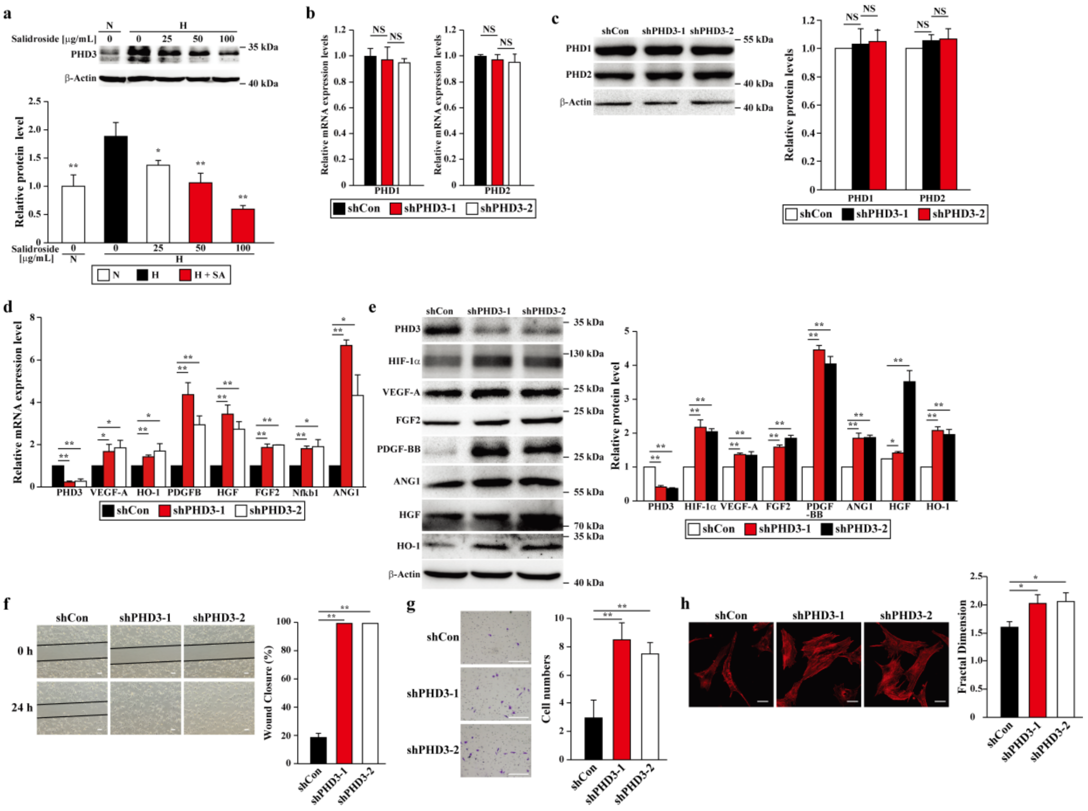

Supplementary Figure 8. PHD3-silencing enhances skeletal muscle cells angiogenic factor expression and mobility potential. (**a**) PHD3 protein expression levels in C2C12 cells treated with indicated concentration of salidroside: (top) representative image and (bottom) the quantitative analysis of western blotting (**p < 0.01 versus cells treated with PBS and cultured in hypoxia). (**b,c**) The mRNA **(b)** and protein **(c)** expression levels of PHD1 and PHD2 in C2C12 cells transfected with shCon, shPHD3-1 or shPHD3-2 and cultured in hypoxic condition were analyzed by using quantitative RT-PCR (NS: not significant, data were shown as relative to the expression in the cells transfected with shCon) and western blotting: representative images (left) and the quantitative analysis (right) (NS: not significant), respectively. (**d,e**) The mRNA **(d)** and protein **(e)** expression levels of angiogenic factors in PHD3-silenced C2C12 cells were analyzed by using quantitative RT-PCR (*p < 0.05, **p < 0.01, data were shown as relative to cells transfected with shCon) and western blotting: representative images (left) and quantification (right) (*p < 0.05, **p < 0.01), respectively. (**f,g**) The mobility of C2C12 cells transfected with shCon, shPHD3-1 or shPHD3-2 in hypoxic condition were analyzed by using scratch assay: (**f**) representative images (left) and percentage of wound closure (right) (**p < 0.01); and (**g**) transwell chamber assay: representative images (left) and quantifiation of migrated cells (right) (**p < 0.01). (**h**) Morphological changes of F-actin were examined by using phalloidin staining: (left) representative images; (right) fractal dimension analysis (*p < 0.05). Scale bars: 100 μm (scratch and transwell chamber assays) or 25 μm (phalloidin staining). N: normoxia, H: hypoxia, SA: salidroside. The quantitative results (**a, b, c, d, e, f, g and h**) are the means ± s.e.m of three independent experiments.

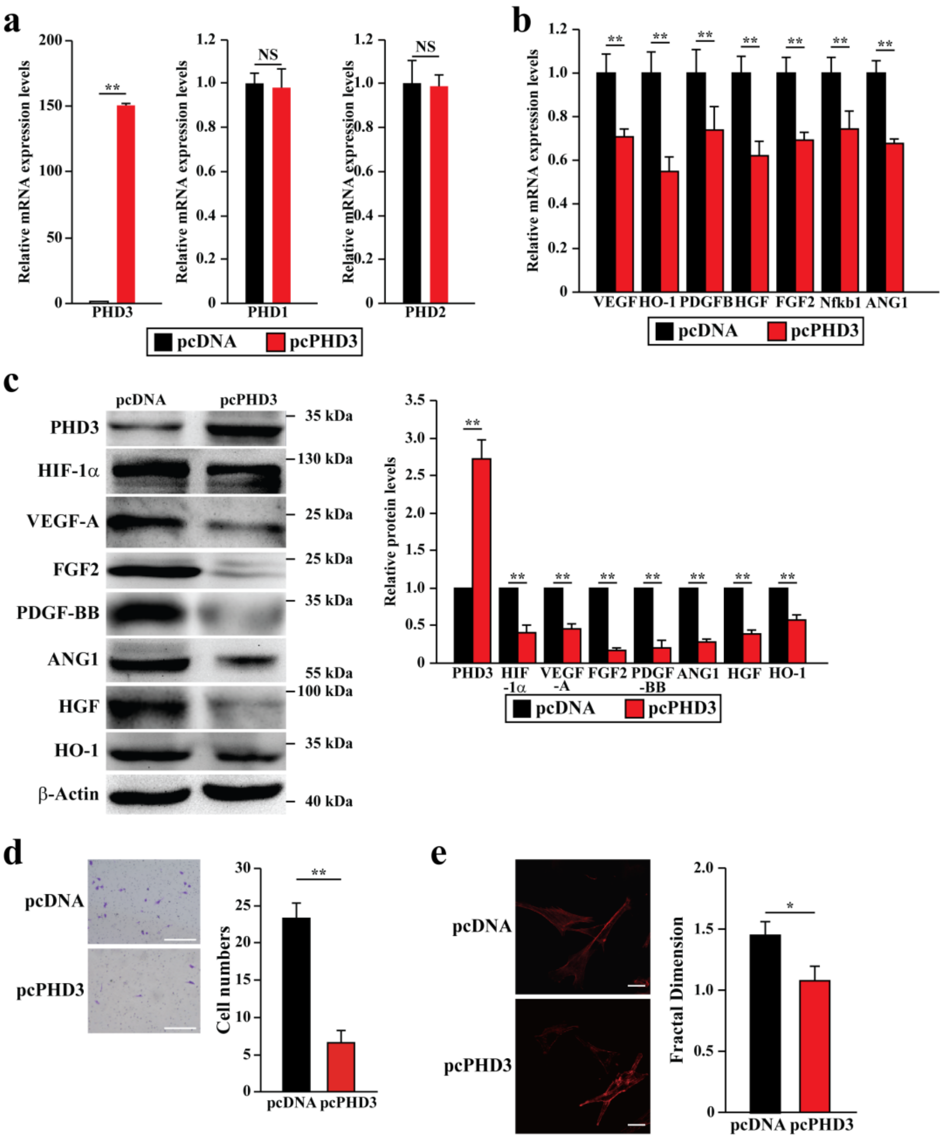

Supplementary Figure 9. PHD3 suppresses skeletal muscle cells angiogenic factor expressions and mobility potential. (**a,b**) The mRNA expression levels of PHDs family **(a)** and angiogenic factors **(b)** in C2C12 cells transfected with PHD3 overexpression vector (pcPHD3) were analyzed by using quantitative RT-PCR (NS: not significant, $^{**}p < 0.01$, data were shown as relative to cells transfected with pcDNA3). (**c**) The protein expression levels of PHD3 and angiogenic factors were analyzed by using western blotting: representative images (left) and the quantitative analysis (right) ($^{**}p < 0.01$), respectively. (**d**) The mobility of C2C12 cells transfected with pcDNA and pcPHD3 in hypoxic condition were analyzed by using transwell chamber assay: representative images (left) and quantifiation of migrated cells (right) ($^{**}p < 0.01$). (**e**) Morphological changes of F-actin were examined by using phalloidin staining: representative images (left) and fractal dimension analysis (right) ($^{*}p < 0.05$). Scale bars: 100 μm (transwell chamber assays) or 25 μm (phalloidin staining). pcDNA: pcDNA3.1(+). The quantitative results (**a, b, c, d and e**) are the means ± s.e.m of three independent experiments.

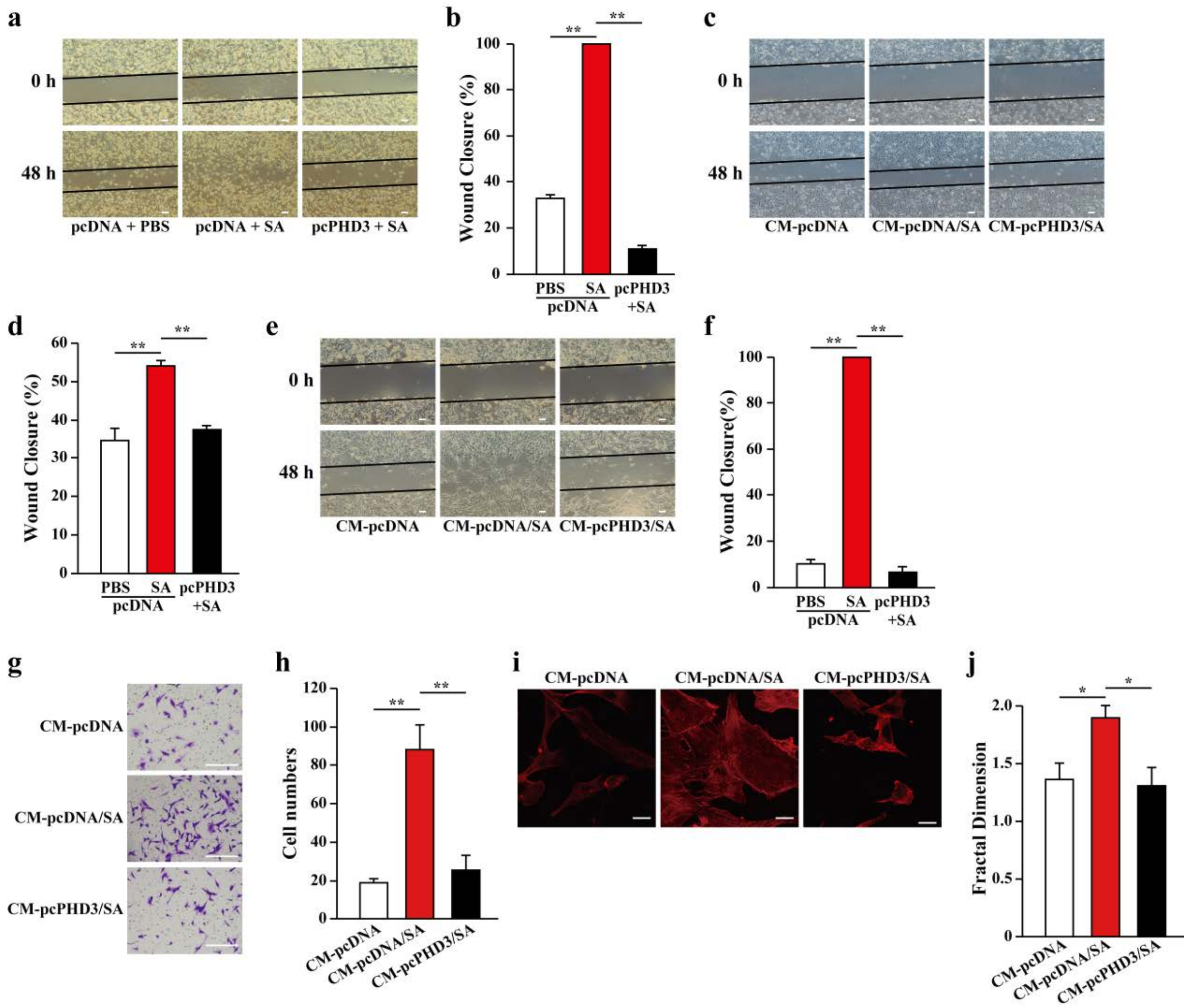

Supplementary Figure 10. Salidroside promotes the paracrine function of skeletal muscle cells through specific inhibition of PHD3. (**a,b**) The mobility of PHD3-overexpressing C2C12 cells treated with salidroside were analyzed by using scratch assay:(**a**) representative images and (**b**) quantification of scratch assay images (**p < 0.01); (**c,d**) The mobility of HUVECs cultured with CM-pcPHD3/SA were analyzed by using scratch assay: (**c**) representative images and (**d**) quantification of scratch assay images (**p< 0.01); (**e-h**) The mobility of MOVAS cells cultured with CM-pcPHD3/SA were analyzed by using: (**e,f**) scratch assay: (**e**) representative images and **(f)** percentage of wound closure (**p < 0.01); (**g,h**) transwell chamber assay: (**g**) representative images and (**h**) percentage of migrated cells (**p < 0.01); (**i,j**) Morphological changes of F-actin were examined by using phalloidin staining: **(i)** representative images; **(j)** quantification analysis of fractal dimension (*p < 0.05). Scale bars: 100 μm (**a, c, e and g**) or 25 μm (**i**). CM-pcPHD3/SA: conditioned medium from pcPHD3-transfected C2C12 cells treated with salidroside. CM-pcDNA and CM-pcDNA/SA: conditioned medium from pcDNA3.1(+)-transfected C2C12 cells treated with PBS or salidroside, respectively. All experiments were done under hypoxic condition. pcDNA: pcDNA3.1(+). The quantitative results (**b, d, f, h and j**) are the means ± s.e.m of three independent experiments.

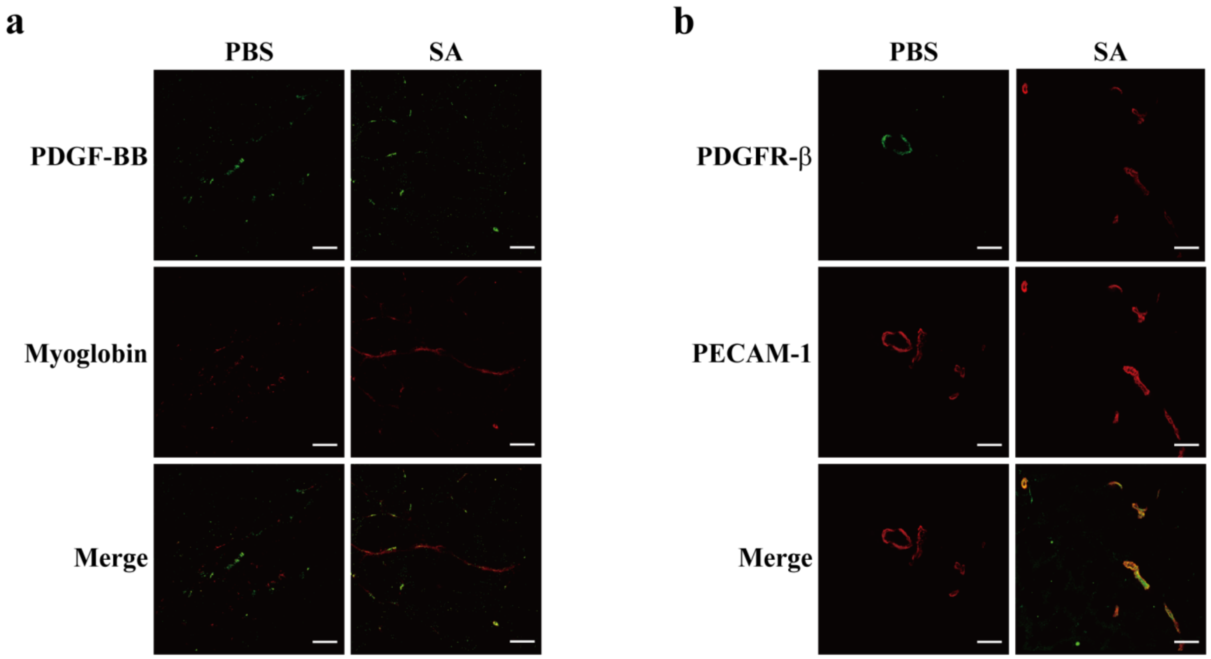

Supplementary Figure 11. Immunohistochemistry of PDGF-BB and PDGFR-β in the gastrocnemius muscle. (**a**) Immunohistochemistry against PDGF-BB (green) and myoglobin (red) in the gastrocnemius muscle of the ischemic hind limbs of HLI mice treated by either salidroside or PBS at 21 days post-surgery was performed by using serial sections (thickness: 4 μm). (**b**) Immunohistochemistry against PDGFR-β (green) and PECAM-1 (red) in the gastrocnemius muscle of the ischemic hind limbs of HLI mice treated by either salidroside or PBS at 21 days post-surgery. SA: salidroside. Scale bars: 100 μm.

Figure 2d

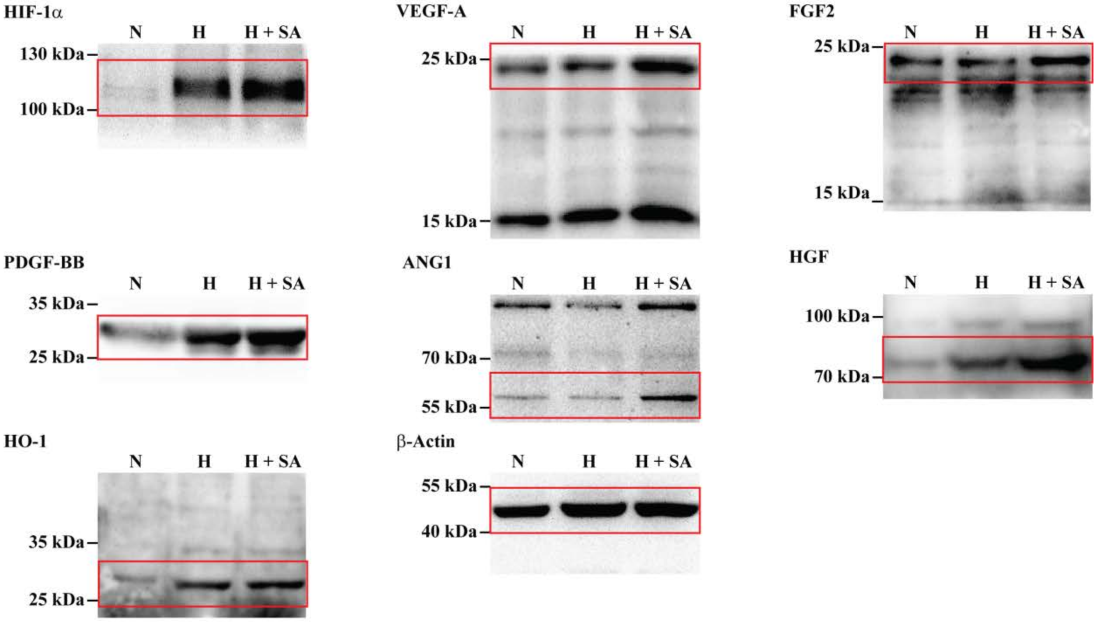

Figure 2g

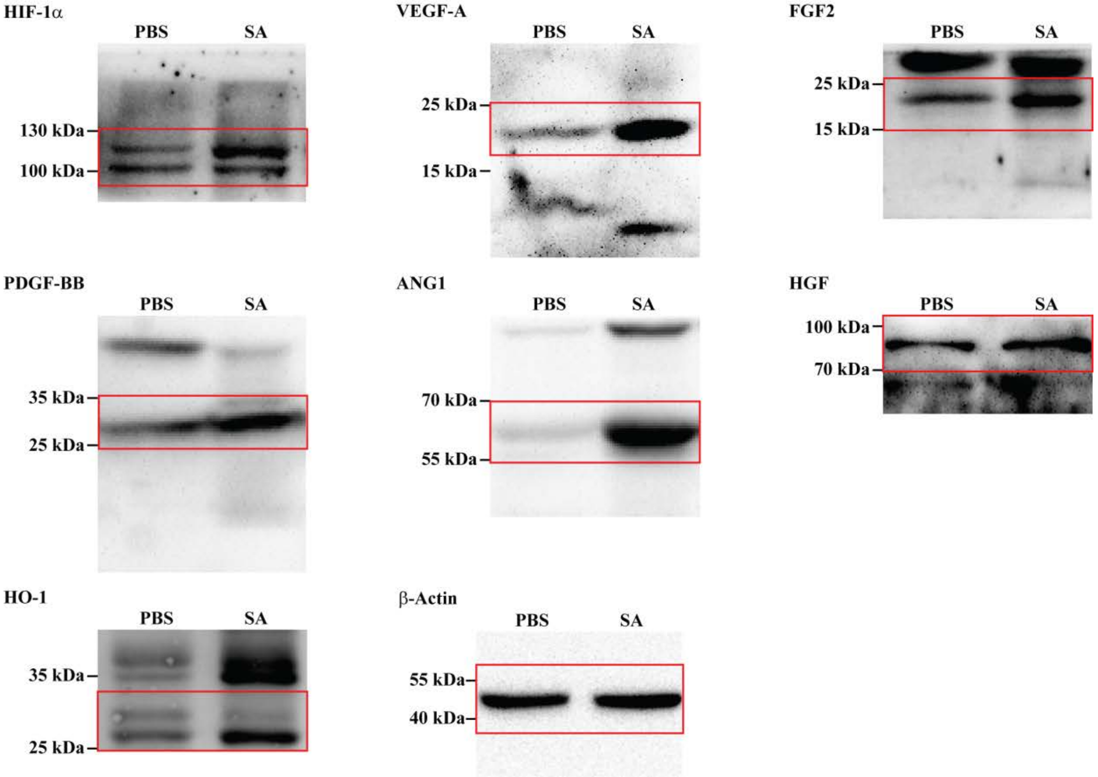

Supplementary Figure 12. Uncropped western blot images of Figure 2. N: normoxia, H: hypoxia, SA: salidroside.

Figure 6b

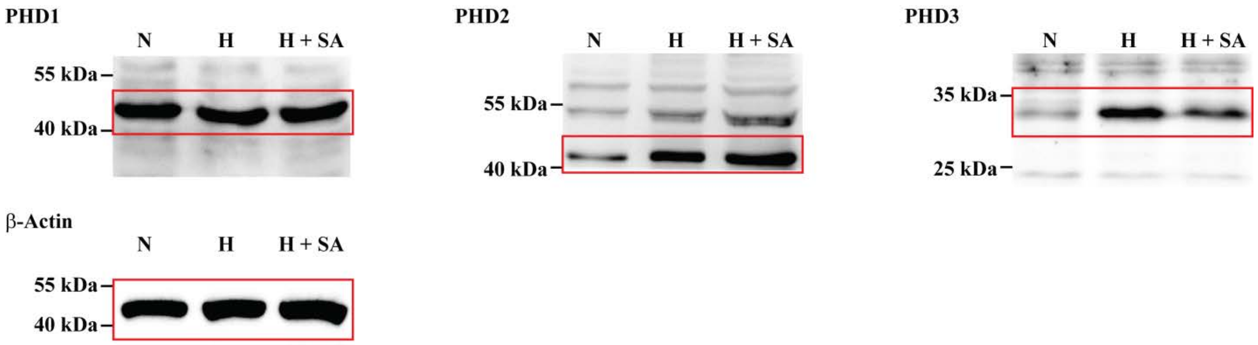

Figure 6d

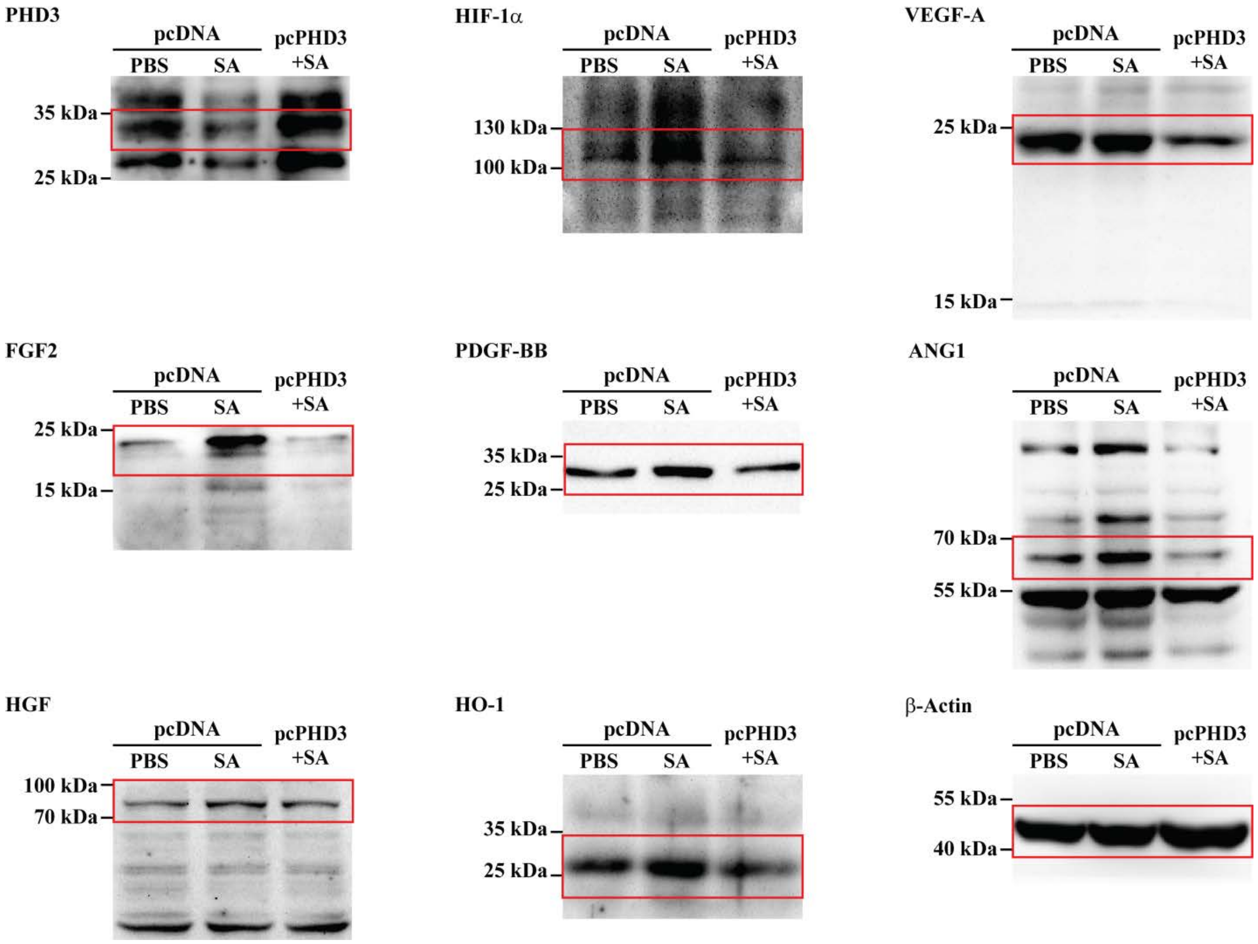

Supplementary Figure 13. Uncropped western blot images of Figure 6. N: normoxia, H: hypoxia, SA: salidroside, pcDNA: pcDNA3.1(+).

Figure 7b

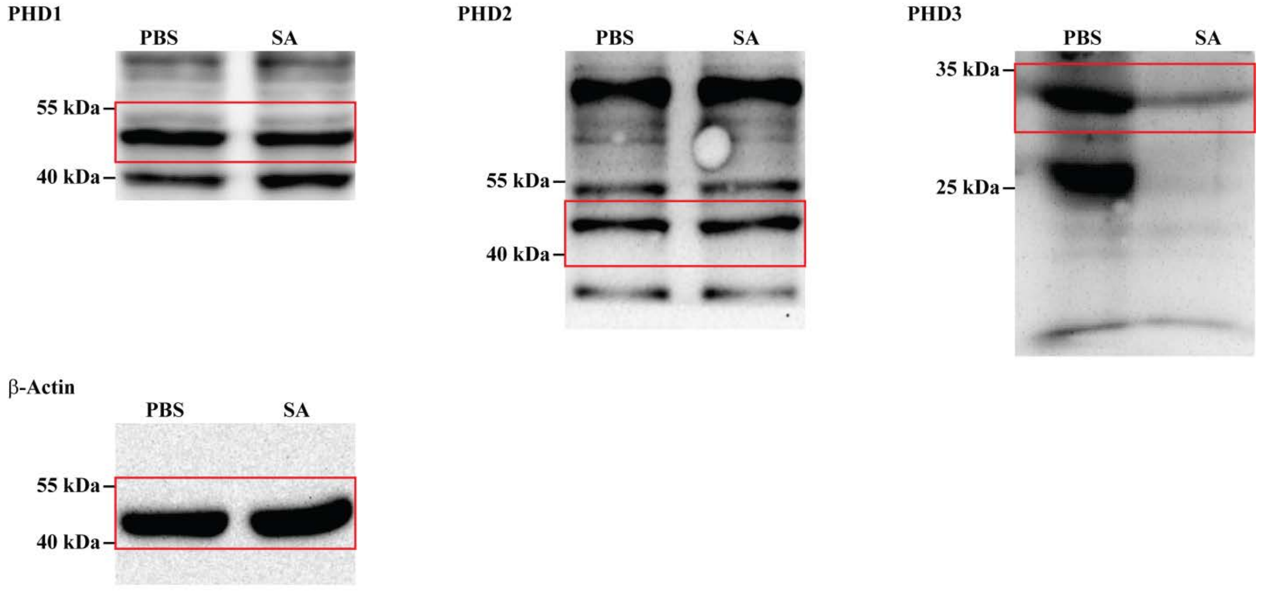

Figure 7i

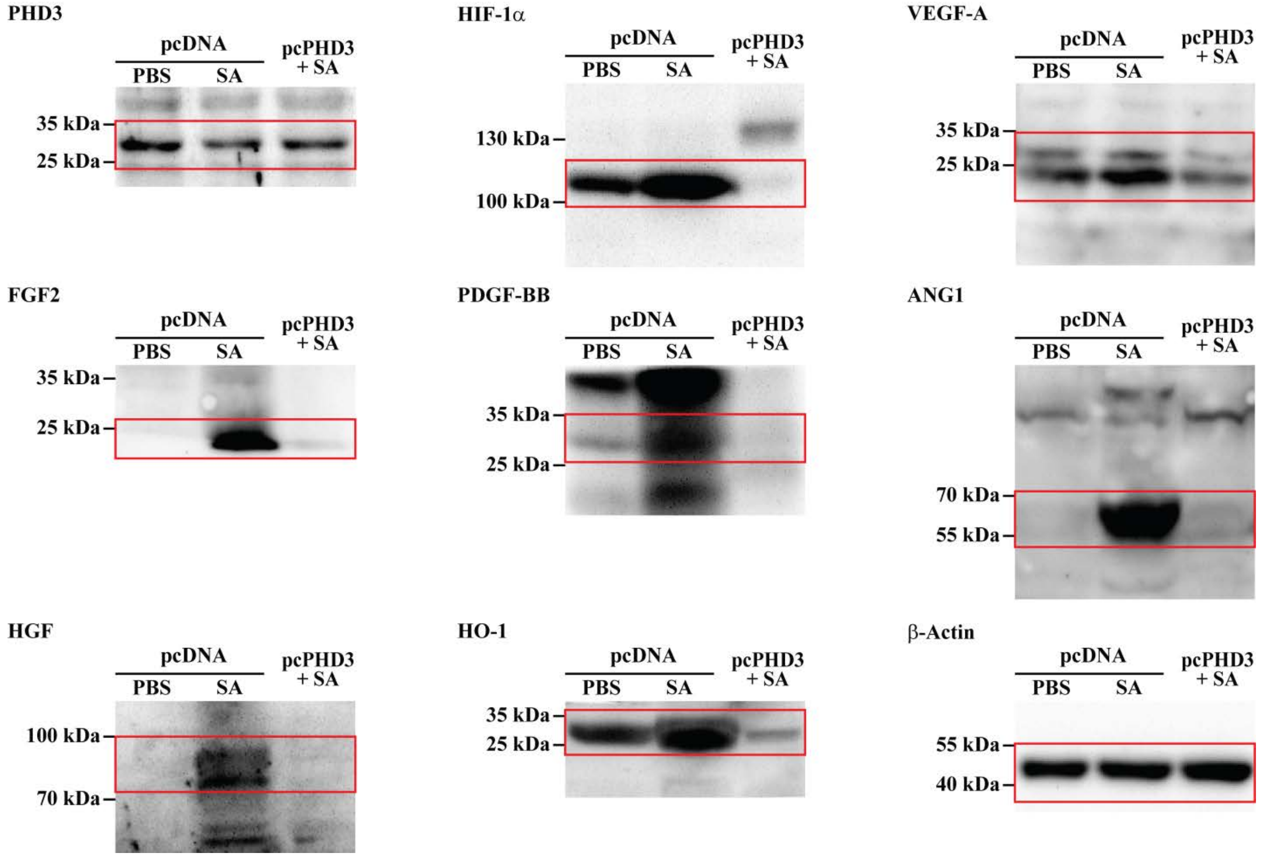

Supplementary Figure 14. Uncropped western blot images of Figure 7. SA: salidroside, pcDNA: pcDNA3.1(+).

Figure 8c

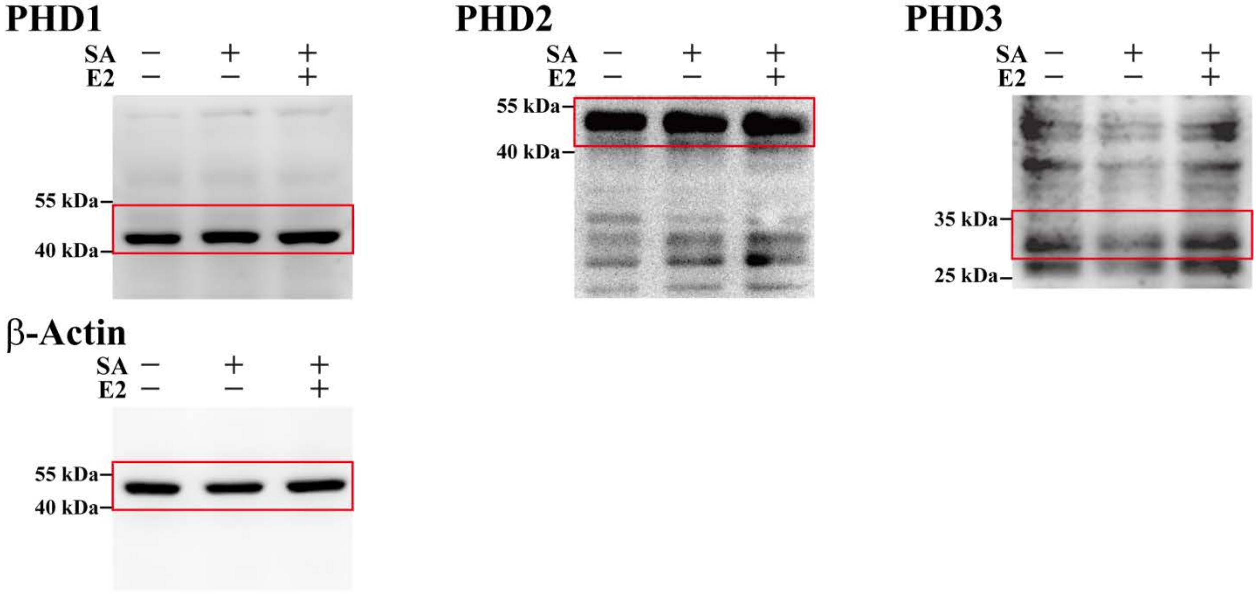

Figure 8d

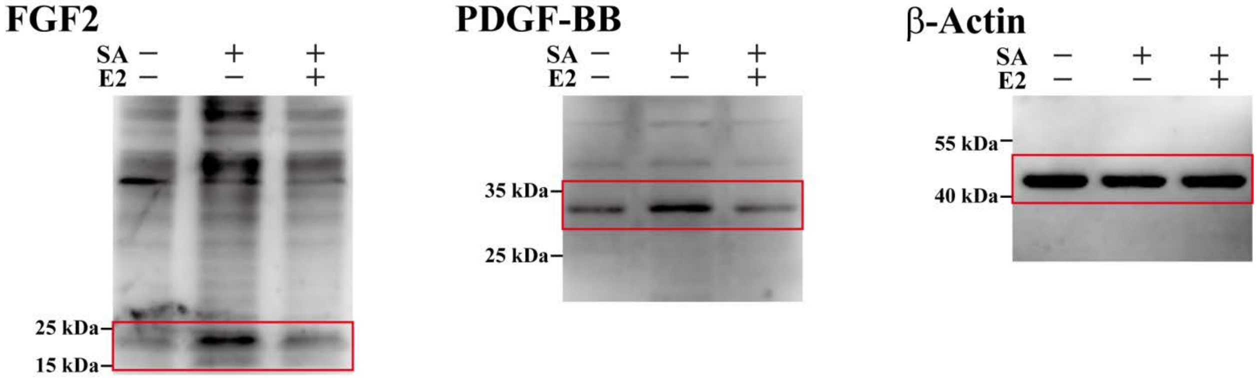

Supplementary Figure 15. Uncropped western blot images of Figure 8. SA: salidroside, E2: 17-β estradiol.

Supplementary Fig. 8a

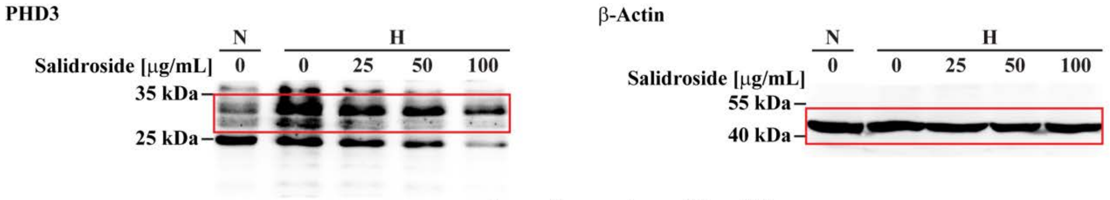

Supplementary Fig. 8d

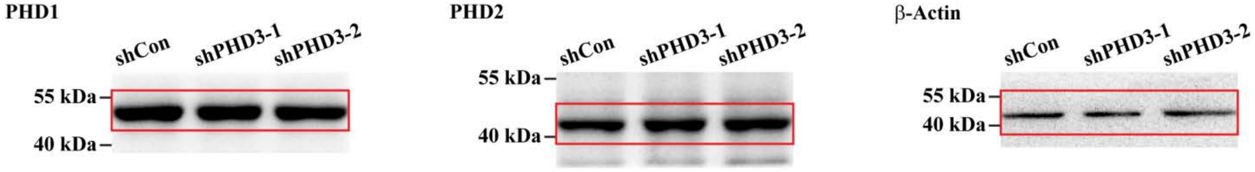

Supplementary Fig. 8f

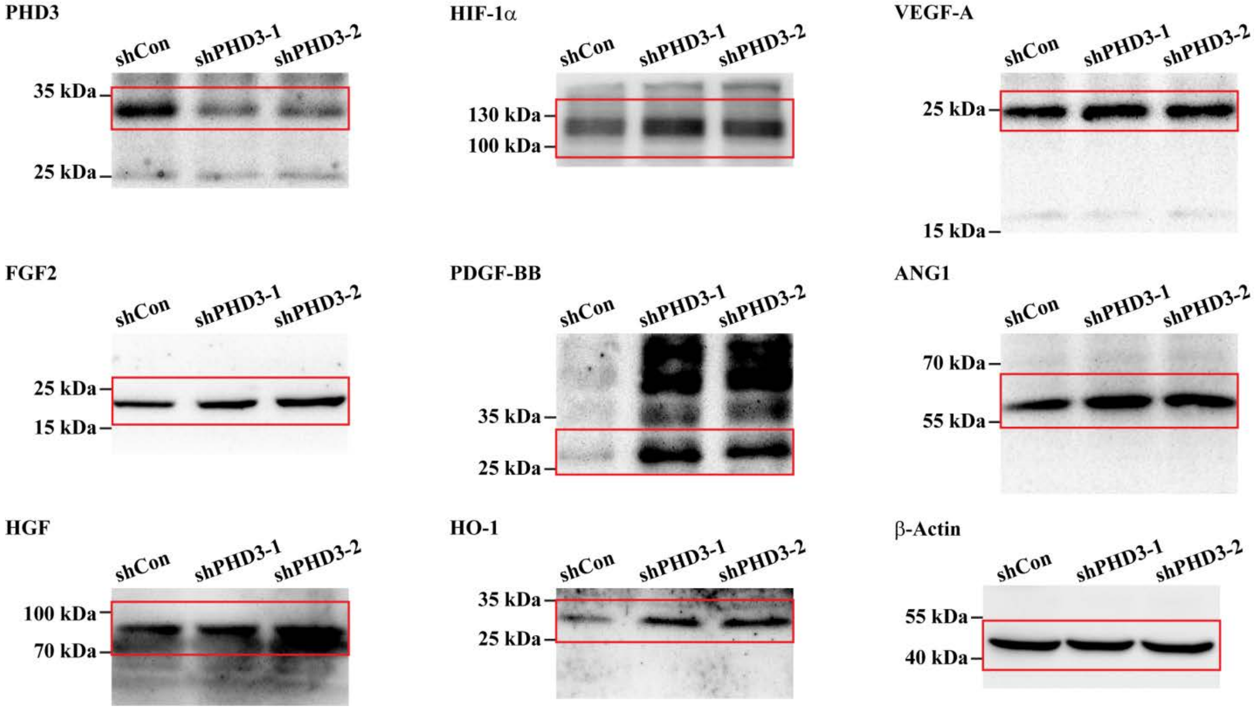

Supplementary Figure 16. Uncropped western blot images of Supplementary Figure 8. N: normoxia, H: hypoxia.

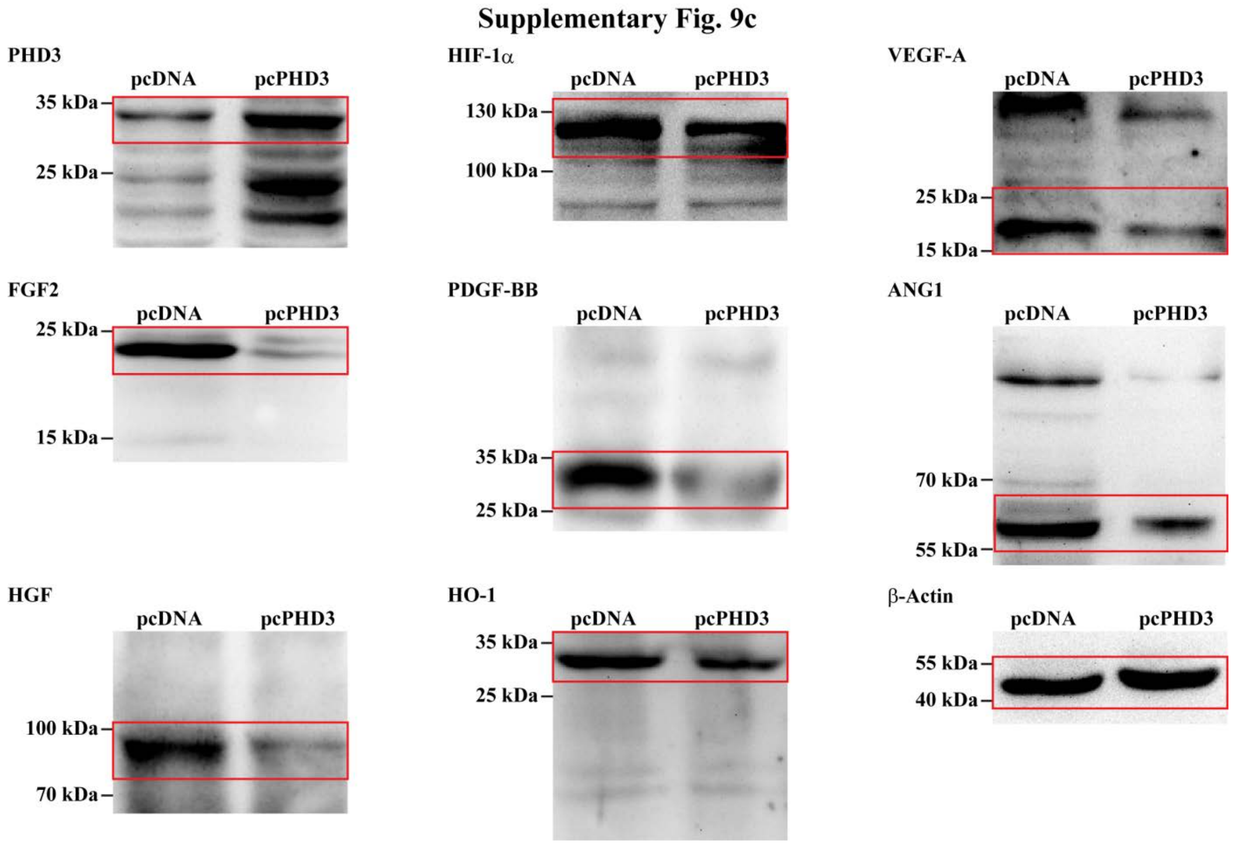

Supplementary Figure 17. Uncropped western blot images of Supplementary Figure 9. pcDNA: pcDNA3.1(+).

Gene	RefSeq No.	Forward	Reverse
VEGF-A	NM_001025257	GCAGAAGTCCCATGAAGTGAT	GTCTCAATCGGACGGCAGTAG
HO-1	NM_010442	AAGAGGCTAAGACCGCCTTC	CATCTGTGAGGGACTCTGGTC
PDGFB	NM_011057	AGCAGAGCCTGCTGTAATCG	GGCTTCTTTCGCACAATCTC
HGF	NM_010427	TGAATGAGTCTGAGTTATGTGC	GAACAATGACACCAAGAACC
FGF2	NM_008006	GTCACGGAAATACTCCAGTTGGT	CCCGTTTTGGATCCGAGTT
Nfkb1	NM_008689	GACCACTGCTCAGGTCCACT	TGTCACTATCCCGGAGTTCA
ANG1	NM_009640	TTGTGATTCTGGTGATTGTGG	CTTGTTTCGCTTTATTTTTGT
β-Actin	NM_007393	AGATGTGGATCAGCAAGCAG	GCGCAAGTTAGGTTTTGTCA
PHD1	NM_053208.4	GGAACCCACATGAGGTGAAG	ACCTTTCTGTCCCGATGCT
PHD2	NM_053207.2	GAAGCTGGGCAACTACAGGA	CATGTCACGCATCTTCCATC
PHD3	NM_028133.2	CAGGTTATGTTCGCCATGTG	CAGGACCCCTCCGTGTAAC
PI3K-1	NM_001024955.2	CACCCAAGCCCACTACTGTA	GAGTGTAATCGCCGTGCATT
EPO	NM_007942.2	TCTGCGACAGTCGAGTTCTG	CTTCTGCACAACCCATCGT
PDGFR-β	NM_001146268.1	TGATGAAGGTCTCCCAGAGG	CTGCTTGCTGTGGCTCTTCT
FGF2R	NM_010206.3	GGGTCATCGAATGGACAAGC	GGGAAAGCTGGGTGAGTACT

Supplementary Table 1. Primer pairs used for quantitative RT-PCR.

Antibody	Product number	Maker	Experiment	Dilution
anti-HIF-1α	NB100-449	Novus Biological	Western Blotting	1/2000
anti-PHD1	NB100-310	Novus Biological	Western Blotting	1/1000
anti-PHD2	NB100-138	Novus Biological	Western Blotting	1/2000
anti-PHD3	NB100-139	Novus Biological	Western Blotting	1/500
anti-VEGF-A	sc-152	Santa Cruz Biotechnology	Western Blotting	1/500
anti-HO-1	sc-10789	Santa Cruz Biotechnology	Western Blotting	1/500
anti-PDGF-BB	sc-7878	Santa Cruz Biotechnology	Western Blotting	1/200
anti-HGF	sc-7949	Santa Cruz Biotechnology	Western Blotting	1/200
anti-FGF2	sc-79	Santa Cruz Biotechnology	Western Blotting	1/500
anti-ANG1	AB10516	Millipore	Western Blotting	1/1000
anti-PDGF-BB	AB23914	Abcam	Immunohistochemistry	1/100
anti-Myoglobin	#164048-1-AP	Protein Tech	Immunohistochemistry	1/200
anti-PDGFR-β	#13449-1-AP	Protein Tech	Immunohistochemistry	1/200
anti-β-Actin	#4967	Cell Signaling Technology	Western Blotting	1/10000
Goat Anti-Rabbit IgG	ZB2301	ZSGB-BIO	Western Blotting	1/10000
anti-PECAM-1	550274	BD Pharmingen	Immunohistochemistry	1/100
Monoclonal anti-murine α-SMA Cy3 conjugate	C6198	Sigma-Aldrich	Immunohistochemistry	1/100
anti-Ki67	Ab15580	Abcam	Immunofluorescence	1/300
Alexa Fluor 488 Donkey Anti- rabbit IgG	A21206	Invitrogen	Immunofluorescence	1/500
Alexa Fluor 488 Goat Anti-Rat IgG	A11006	Invitrogen	Immunohistochemistry	1/500
Alexa Fluor 688 Goat Anti-Rabbit IgG	A0468	Beyotime	Immunohistochemistry	1/100
Alexa Fluor 568 Goat Anti-Rat IgG	A11077	Invitrogen	Immunohistochemistry	1/500
Phalloidin	A34055	Invitrogen	Immunofluorescence	1/250
DAPI	C1006	Beyotime	Transwell chamber assay Immunofluorescence	not diluted

Supplementary Table 2. Antibodies used for western blotting, immunohistochemistry, immunofluorescence and transwell chamber assay.

Supplementary Methods

RNA extraction and quantitative RT-PCR analysis

Total RNA from gastrocnemius muscles or cells was extracted with Trizol (Invitrogen) according to the manufacturer's instruction. Total RNA (1 μg) was reverse-transcribed into cDNA using the PrimeScript RT Reagent Kit with gDNA Eraser (Takara Bio), and quantitative RT-PCR was performed to assess the mRNA expression levels with SYBR Premix Ex Taq (Takara Bio). The sequences of the primers used for quantitative RT-PCR were shown in Supplementary Table 1. β-actin was used to normalize sample amplifications. The results were shown as relative to the expression level in the corresponding controls, which are assumed as 1.

Western blotting

For cell culture experiments, cells were collected and lysed with RIPA lysis buffer with protease inhibitor and phosphatase inhibitor cocktail (complete cocktail; Roche Applied Science, Mannheim, Germany). For mouse experiments, the gastrocnemius muscle was isolated and immediately homogenized with RIPA lysis buffer with protease inhibitor and phosphatase inhibitor cocktail (complete cocktail; Roche Applied Science) to obtain protein extracts. Equal amounts of the sample proteins were electrophoresed on sodium dodecyl sulfate polyacrylamide gel and transferred to a polyvinylidene fluoride (PVDF) membrane (Millipore, Billerica, MA). The antibodies used are listed in Supplementary Table 2, and immunoblotting with anti-β-actin antibody was conducted to ensure equal protein loading. The signal was measured by the SuperSignal West Femto Maximum Sensitivity Substrate detection system (Thermo Scientific, Waltham, MA). The quantitative analysis was performed by using Quantity One (Thermo Scientific), and β-actin

was used as an internal control. The results were shown as relative to the expression level in the corresponding controls, which are assumed as 1.

Immunohistochemical analysis

Frozen gastrocnemius muscles were sectioned at 10 μm thickness using a cryostat and subjected to immunohistochemistry. Briefly, for immunohistochemistry against PECAM-1 and α-SMA, the frozen muscle tissue sections were incubated with a primary antibody against murine PECAM-1 for 1 h. The specimens were then incubated with a monoclonal antibody against murine α-SMA conjugated with Cy3 and Alexa Fluor 488 Goat Anti-Rat IgG (Invitrogen) for 1 h at room temperature. For immunohistochemistry against PECAM-1 and PDGFR-β, the frozen muscle tissue sections were incubated with a primary antibody against murine PDGFR-β for 90 min prior to incubation with Alexa Fluor 488 Goat Anti-Rat IgG (Invitrogen) for 90 min at room temperature. Then the sections were further incubated with a primary antibody against murine PECAM-1 for 90 min followed by incubation with Alexa Fluor 568 Goat Anti-Rat IgG (Invitrogen) for 90 min at room temperature. For immunohistochemistry against PDGF-BB and myoglobin, serial sections at 4 μm thickness were obtained from the frozen muscle tissue and incubated with a primary antibody against murine PDGF-BB or myoglobin for 90 min prior to incubation with Alexa Fluor 488 Donkey Anti-Rabbit IgG (Invitrogen) or Alexa Fluor 688 Goat Anti-Rabbit IgG (Beyotime) for 90 min at room temperature. Images were taken with Microsystems-AF6000 (Leica, Germany). The antibodies used were listed in Supplementary Table 2.

Immunofluorescence staining

Cells were seeded in 3.5-cm culture dishes (5 × 10^4 cells per dish), treated with salidroside (final concentration: 100 μg/ml) or PBS for 24 h, washed, and incubated under hypoxia for 24 h. Then, the cells were fixed for 30 min at room temperature with 4% paraformaldehyde, permeabilized for 5 min with PBS containing 0.1% Triton X-100, blocked with 1% BSA for 1 h, and incubated with antibody against Ki67 for 1 h or phalloidin for 30 min (Supplementary Table 2). The nuclei were stained with DAPI (C1006, Beyotime, China) for 15 min. Images were taken with Microsystems-AF6000 for Ki67, and Microsystems-TCS SP5 (Leica), and quantification was performed by using LASX software (Leica). For experiments with conditioned media, the cells were cultured with conditioned medium under hypoxia for 24 h prior to staining.

Scratch assay

Cells were seeded in 6-well plates at a density of 3 × 10^5 cells per well, treated with salidroside (final concentration: 100 μg/ml) or PBS for 24 h under normoxic condition, and washed with PBS. After the medium was changed to culture medium without salidroside, cells were treated with cyclohexamine (purity ≥ 95%, final concentration: 10 mg/ml, Cayman Chemicals, USA), then a scratch wound was created with a micropipette tip, and the cells were incubated under normoxia or hypoxia for the indicated times. For experiments with conditioned media, cells were cultured with conditioned medium under hypoxia after the scratch wound was created for the indicated times. For experiments using FGF2R and PDGFR inhibitors, cells were cultured with conditioned media and PD173074, CP868596 or both of them under hypoxia after the scratch wound was created for the indicated times.

Transwell chamber assay

Cells were treated with PBS or salidroside (final concentration: 100 μg/ml) and incubated under normoxic condition for 24 h. The cells were reseeded (5×10^3 cells per chamber) in the upper chambers of a transwell plate (Corning, NY, USA) and cultured under hypoxia for 24 h. Normal culture medium was placed in the lower chambers. Cells migrated to lower chamber were stained with crystal violet (Beyotime) for imaging or DAPI (Beyotime) for quantification by averaging the number of the cells in the lower chamber. Images were taken with Olympus IX7I (Japan). For experiments with conditioned media, cells were seeded (5×10^3 cells per chamber) in the upper chambers of a transwell plate (Corning) and cultured under hypoxia for 24 h. Conditioned media were placed in the lower chambers. For experiments using FGF2R and PDGFR inhibitors, conditioned media and PD173074, CP868596 or both of them were placed in the lower chambers.

Enzyme-linked immunosorbent assay (ELISA)

The amounts of FGF2 and PDGF-BB in the culture medium were measured using a commercially available FGF2 and PDGF-BB sandwich ELISA kit (CK-E91925M and CK-E9420M, respectively, Yuanye, Shanghai, China) according to the manufacturer's protocol.

Hematoxylin and Eosin staining

The gastrocnemius muscles were fixed with 4% paraformaldehyde for overnight prior to being embedded in paraffin and sectioned at 5 μm thickness using a cryostat. Sections were dewaxed using xylene and rehydrated before being stained with Hematoxylin and Eosin (Beyotime).

High performance liquid chromatography-mass spectrometry (HPLC-MS) analysis

Samples were collected from: (1) culture medium (DMEM supplemented with 10% FBS); (2) standard reference of salidroside; (3) culture medium collected from C2C12 cells cultured with DMEM supplemented with 10% FBS and salidroside (final concentration 100 μg/ml) for 24 h in normoxia; (4) culture medium collected from C2C12 cells cultured with DMEM supplemented with 10% FBS and salidroside (final concentration 100 μg/ml) for 24 h in normoxia, washed with PBS twice, added with fresh DMEM supplemented with 10% FBS and cultured in hypoxia for 0 h; (5) culture medium collected from C2C12 cells cultured with DMEM supplemented with 10% FBS and salidroside (final concentration 100 μg/ml) for 24 h in normoxia, washed with PBS twice, added with fresh DMEM supplemented with 10% FBS and cultured in hypoxia for 24 h (*i.e.*, CM-SA). Analysis for salidroside carryover was performed by using HLMS (6120 Quadrupole LC/MS, Agilent Technologies, CA, America). Data were processed using Agilent IO Libraries Suite 16.3 (Agilent Technologies). The injection volume is 20 μl and the retention time is 1.67-1.73 minute. Gradient elution was performed using solvent A (DI water + 0.1% formic acid) and solvent B (acetonitrite + 0.1% formic acid), and the gradient flow was as follows: 0 min 10.0% B, 5 min 95.0% B, 7.5 min 95% B. The flow rate was 1 ml/min.
